# Combined social communication therapy at home and in education for young autistic children in England (PACT-G): a parallel, single-blind, randomised controlled trial

**DOI:** 10.1016/S2215-0366(22)00029-3

**Published:** 2022-04

**Authors:** Jonathan Green, Kathy Leadbitter, Ceri Ellis, Lauren Taylor, Heather L Moore, Sophie Carruthers, Kirsty James, Carol Taylor, Matea Balabanovska, Sophie Langhorne, Catherine Aldred, Vicky Slonims, Victoria Grahame, Jeremy Parr, Neil Humphrey, Patricia Howlin, Helen McConachie, Ann Le Couteur, Tony Charman, Richard Emsley, Andrew Pickles

**Affiliations:** a Division of Neuroscience and Experimental Psychology, University of Manchester, Manchester, UK; b Institute of Education, University of Manchester, Manchester, UK; c Manchester Royal Children's Hospital, Manchester Academic Health Sciences Centre, Manchester, UK; d Department of Psychology, Institute of Psychiatry, Psychology and Neuroscience (IoPPN), King's College London, London, UK; e Department of Biostatistics and Health Informatics, Institute of Psychiatry, Psychology and Neuroscience (IoPPN), King's College London, London, UK; f Population Health Sciences Institute, Newcastle University, Newcastle upon Tyne, UK; g Sir James Spence Institute, Royal Victoria Infirmary, Newcastle upon Tyne, UK; h Evelina London Children's Hospital, Guy's & St Thomas’ NHS Foundation Trust, London, UK; i Complex Neurodevelopmental Disorders Service (CNDS), Cumbria, Northumberland, Tyne and Wear NHS Foundation Trust, Newcastle upon Tyne, UK; j Great North Children's Hospital, Newcastle upon Tyne NHS Foundation Trust, Newcastle upon Tyne, UK

## Abstract

**Background:**

Autistic children can have difficulty generalising treatment effects beyond the immediate treatment context. Paediatric Autism Communication Therapy (PACT) has been successful when delivered in the clinic. Here we tested the Paediatric Autism Communication Therapy-Generalised (PACT-G) intervention combined between home and education settings for its overall effect and mechanistic transmission of effect across contexts.

**Methods:**

In this parallel, single-blind, randomised, controlled trial, we recruited autistic children aged 2–11 years in urban or semi-urban areas in Manchester, Newcastle, and London, England. Children needed to meet core autism criteria on Autism Diagnostic Observation Schedule-second edition (ADOS-2) and parent-rated Social Communication Questionnaire (SCQ-lifetime), and children older than 5 years were included if they had intentional communication but expressive language equivalent of age 4 years or younger. Eligible children were randomly assigned (1:1), using block randomisation (random block sizes of 2 and 4) and stratified for site, age (2–4 years *vs* 5–11 years), and gender, to either PACT-G plus treatment as usual or treatment as usual alone. Research assessors were masked to treatment allocation. The PACT-G intervention was delivered by a therapist in parallel to the child's parents at home and to learning-support assistants (LSA) at their place of education, using both in-person and remote sessions over a 6 month period, to optimise adult–child social interaction. Treatment as usual included any health support or intervention from education or local community services. The primary outcome was autism symptom severity using the ADOS-2, as measured by researchers, at 12 months versus baseline. Secondary outcomes were Brief Observation of Social Communication Change (BOSCC) and dyadic social interaction between child and adult across contexts, both at 12 months. Other secondary outcome measures were assessed using the following composites: language, anxiety, repetitive behaviour, adaptive behaviour, parental wellbeing, child health-related quality of life, and disruptive behaviour. Assessments were done at baseline, 7 months, and 12 months. We used an intention-to-treat (ITT) analysis of covariance for the efficacy outcome measures. Adverse events were assessed by researchers for all trial families at each contact and by therapists in the PACT-G group at each visit. This study is registered with the ISRCTN Registry, ISRCTN 25378536.

**Findings:**

Between Jan 18, 2017, and April 19, 2018, 555 children were referred and 249 were eligible, agreed to participate, and were randomly assigned to either PACT-G (n=122) or treatment as usual (n=127). One child in the PACT-G group withdrew and requested their data be removed from the study, giving an ITT population of 248 children. 51 (21%) of 248 children were female, 197 (79%) were male, 149 (60%) were White, and the mean age was 4·0 years (SD 0·6). The groups were well balanced for demographic and clinical characteristics. In the PACT-G group, parents of children received a median of 10 (IQR 8–12) home sessions and LSAs received a median of 8 (IQR 5–10) education sessions over 6 months. We found no treatment effect on the ADOS-2 primary outcome compared with treatment as usual (effect size 0·04 [95% CI –0·19 to 0·26]; p=0·74), or researcher-assessed BOSCC (0·03 [–0·25 to 0·31]), language composite (–0·03 [–0·15 to 0·10]), repetitive behaviour composite (0·00 [–0·35 to 0·35]), adaptive behaviour composite (0·01 [–0·15 to 0·18]), or child wellbeing (0·09 [–0·15 to 0·34]). PACT-G treatment improved synchronous response in both parent (0·50 [0·36 to 0·65]) and LSA (0·33 [0·16 to 0·50]), mediating increased child communication with parent (0·26 [0·12 to 0·40]) and LSA (0·20 [0·06 to 0·34]). Child dyadic communication change mediated outcome symptom alteration on BOSCC at home (indirect effect –0·78 [SE 0·34; 95% CI –1·44 to –0·11]; p=0·022) although not in education (indirect effect –0·67 [SE 0·37; 95% CI –1·40 to 0·06]; p=0·073); such an effect was not seen on ADOS-2. Treatment with PACT-G also improved the parental wellbeing composite (0·44 [0·08 to 0·79]) and the child disruptive behaviour composite in home and education (0·29 [0·01 to 0·57]). Adverse events on child behaviour and wellbeing were recorded in 13 (10%) of 127 children in the treatment as usual group (of whom four [31%] were girls) and 11 (9%) of 122 in the PACT-G group (of whom three [33%] were girls). One serious adverse event on parental mental health was recorded in the PACT-G group and was possibly study related.

**Interpretation:**

Although we found no effect on the primary outcome compared with treatment as usual, adaptation of the 12-month PACT intervention into briefer multicomponent delivery across home and education preserved the positive proximal outcomes, although smaller in effect size, and the original pattern of treatment mediation seen in clinic-delivered therapy, as well as improving parental wellbeing and child disruptive behaviours across home and school. Reasons for this reduced efficacy might be the reduced dose of each component, the effect of remote delivery, and the challenges of the delivery contexts. Caution is needed in assuming that changing delivery methods and context will preserve an original intervention efficacy for autistic children.

**Funding:**

National Institute for Health Research and Medical Research Council Efficacy and Mechanism Evaluation Award.

## Introduction

The pattern of findings across a number of early childhood interventions for autism is reproducible for moderate-to-good effects on targeted proximal or intermediate outcomes, such as improvement in social interaction and communication measured close to the treatment context.^1^, ^2^, ^3^ However, for an intervention to show a tangible effect on a child's life and overall development, the challenge is to show treatment effects generalised beyond the immediate intervention context—eg, in interaction with others in school or at home, or affecting other developmental, functional, or symptom outcomes over a variety of contexts or over time—for which there is much less evidence.^4^, ^5^ Thus, understanding and improving the transmission of targeted intervention effects that are observed close to the treatment context into functional change in wider contexts of a child's life is a key challenge for autism treatment research.^2^, ^5^

Capacity to generalise acquired skills flexibly across different situations, people, and environmental contexts is central to early development, but has often been suggested as difficult for autistic individuals. Such generalisation has been studied at the level of transfer of discrete behaviours, for which empirical evidence seems inconsistent.^6^ A broader definition would include the ability to apply domains of related skills (eg, in social communication) flexibly across contexts or as a developmental cascade from precursor skills through to developmentally related subsequent abilities or adaptations.^2^, ^4^ Plausible barriers to generalisation in this broader sense include evidence of the autistic child's relatively altered internal symbolic representation or cognitive central coherence, which might interfere with the consolidation and transfer of procedural aspects of skill.

**Research in context**

**Evidence before this study**

We searched Ovid and Scopus (including PubMed and MEDLINE) for publications in English from database inception up to Nov 21, 2021, using the terms Child*.tw. AND (Review OR meta* OR systematic).ti. AND (Randomi* OR RCT OR Generali* OR bias* OR Treatment* OR program*).tw. AND (Auti* OR neurodevelopment* OR neurodisabilit* OR developmental disorder* OR ASD).ti. AND ((Mechan* OR Mediat*).ab. OR (Mechan* OR Mediat*).tw.) AND (Child*.ab. OR child*.tw.) AND ((Randomi* OR Control* OR Trial OR RCT).ti. OR (Randomi* OR Control* OR Trial OR RCT).ab.) AND ((Treatment* OR Therap* OR Intervention* OR Training).ti. OR (Treatment* OR Therap* OR Intervention* OR Training).ab. OR (Treatment* OR Therap* OR Intervention* OR Training).tw.). Narrative and systematic reviews in the past 5 years, which have selected for study quality, found that early interventions for autism can often show improvement in proximal outcomes close to the therapy context, with a small number of mechanism studies suggesting that aspects of dyadic interaction (ie, joint engagement, caregiver responsiveness, and synchrony) can affect child dyadic function. However, demonstration of the generalisation of these effects across contexts and people or over time into autism-specific symptoms or child adaptation has rarely been shown, and only one mediation study to date has investigated the mechanism for such generalisation. Largely clinic-based delivery of the PACT model had shown reduction in autism symptom severity, which was sustained through development over time in follow-up and mediated by treatment effects on the proximal intervention targets of adult–child dyadic social communication.

**Added value of this study**

In this randomised controlled trial in parallel home and education settings in England, we found that autism symptom outcomes in home, education, or research settings were not affected compared with treatment as usual alone. The multicomponent social communication PACT-G intervention with parents at home and learning-support assistants resulted in similar proximal positive effects as had PACT, although with a smaller effect size. It also showed the same mediation pathway from adult response to child communication in the dyad. We found positive treatment effects on parental wellbeing and child disruptive behaviour in home and education.

**Implications of all the available evidence**

Generalised symptom reduction outcomes were not observed with this intervention implemented in education and at home; however, proximal intervention effects can be achieved with such parallel delivery. For educational settings, curriculum-embedded interventions (rather than social-communication interventions implemented in an education context) might show better effects, although the available evidence base is small. Our study suggests caution in assuming preserved outcome efficacy for interventions in autistic children when the mode of treatment delivery is changed. Future research should investigate mechanism of effects and delivery mode-specific investigations of efficacy.

Previous trials of the Paediatric Autism Communication Therapy (PACT), a parent-mediated therapy, showed a substantial effect on the targeted proximal dyadic effects of parental communicative synchrony with the child^7^, ^8^ and, independently, child's communication initiations with the parent.^8^ Generalisation of these proximal dyadic interaction effects across contexts into change on a standardised objective child autism assessment was significant in one trial^7^ and non-significant in another,^8^ but was significant when social communication, restricted repetitive behaviours, and sensory symptoms were combined in the full autism phenotype.^9^ This effect then persisted through 6 years of follow-up, as the child developed, to give an overall treatment effect size on symptoms during treatment and follow-up of 0·55 (95% CI 0·14–0·91).^9^ Mediation analysis supported previous work^10^ in confirming a causal chain of effect; first, within the dyad, from the increased parental synchrony targeted by treatment to improved child communication initiation, and second, within the child, from their improved dyadic communication with their parent to improved autism symptoms with researcher at endpoint.^11^ These two transmission steps are probably subject to different mechanisms; the first related to the interpersonal dynamics of dyadic interaction, the second to within-child mechanisms for generalisation of acquired change across behaviour domains, contexts and people, and through time.

Here we report on the Paediatric Autism Communication Trial-Generalised (PACT-G) randomised controlled trial, in which we aimed to reinforce and amplify this second generalisation step.^12^ The PACT intervention was adapted into a multicomponent simultaneous intervention in home and education. By working in these two naturalistic settings, we aimed to support the child's consolidation and transfer of procedural aspects of skill across context, which were identified as a plausible barrier to generalisation. Additionally, the PACT-G intervention was extended to include children older than 5 years up to age 11 years who had continuing communication impairments. Autism intervention studies to date have been largely limited to preschool (ie, aged <5 years) interventions; however, early communication development continues into the early school years (up to age 11 years)^13^ and social communication skills in that period are strong predictors for later development.^14^ The persisting and substantial impairments in social interaction and communication among autistic children support a developmentally sustained intervention into middle childhood (age 5–11 years), using the child's naturalistic learning environments.

In this Article, we use identity-first language rather than person-first language; we acknowledge and respect the different views on this use within the autism community and among professionals.

## Methods

### Study design and participants

In this parallel, single-blind, randomised, controlled trial, participants were recruited at three sites in England (Manchester, Newcastle, and London) after referral via clinical specialists and education professionals.

We included clinically diagnosed children aged 2–11 years meeting core autism criteria on Autism Diagnostic Observation Schedule-second edition (ADOS-2)^15^ and parent-rated Social Communication Questionnaire (SCQ-lifetime);^16^ children older than 5 years were included if they had intentional communication but the expressive language of an individual aged 4 years or younger, or equivalent. Exclusion criteria included: children with a younger than 12 months non-verbal age-equivalent level, parents who do not speak English at home, absence of agreement from child's education setting. Detailed inclusion criteria are in the appendix (p 1). Data were collected on self-reported gender provided by the parent for themselves and their child. We did not collect gender for learning-support assistants (LSAs). The trial took place in family homes, mainstream nursery and preschool settings, specialist nurseries, mainstream schools with specialist autism units, and specialist autism school settings. Clinical or educational teams who initially introduced the trial to parents and interested families then referred them to the research team for information and consent. Written informed consent was obtained from nominated trial parents for the participation of both themselves and their child. Child verbal assent was obtained directly or established through observations of affect and behaviour.

Ethical approval for this trial was granted from the North West-Greater Manchester Central Research Ethics Committee on Jan 28, 2016 (reference 15/NW/0912). The trial protocol is in the appendix (pp 22–42).

### Randomisation and masking

After baseline assessment, children were randomly assigned (1:1) to either PACT-G plus treatment as usual or treatment as usual alone. Randomisation was done via a web-based service hosted by King's Clinical Trial Unit (London, UK) with password-protected access only to trained trial staff. Randomisation was at the level of the individual participant, stratified by site, age group (2–4 years *vs* 5–11 years), and gender, using random block sizes (random block sizes of 2 and 4). Once randomised, the system automatically generated an unblinded email confirmation that was sent to the therapy lead at each site and a blinded copy to the researcher who made the request and to the trial manager. Researchers who administered assessments were masked to participant group allocation and every effort was made to maintain masking throughout the trial. Research staff were located separately from therapists. To preserve masking in assessment, parents were reminded at every meeting with researchers about the importance of not divulging treatment group allocation. Education settings were given photographs to distinguish therapists from research staff, and, where possible, different staff signed-in therapists and researchers. To preserve masking on follow-up visits, Dyadic Communication Measure for Autism (DCMA), Brief Observation of Social Communication Change (BOSCC), and ADOS-2 assessments were cross-coded from videotapes by non-administering researchers. All other researchers who did assessments were also masked to treatment group assignment. Trial statisticians were masked to treatment allocation (with dummy variables for groups) until the last stage of the analysis after data lock.

Participant families and LSAs could not be masked to treatment group allocation. All therapy sessions were videotaped, and variability due to therapist effects was minimised via frequent clinical supervision and checks on continuing therapist fidelity against the treatment manual. Randomly selected sessions for each therapist were formally coded for fidelity over the course of the study by independent clinicians using a model that has been successfully used previously.^8^

### Procedures

The PACT-G intervention is an adaptation into home and education contexts of the original clinic-delivered PACT.^12^ It is a caregiver-mediated intervention in which therapists use video feedback of caregiver–child interaction to enhance caregiver awareness of and synchronous response to child social communication^17^ in a way that has been shown to increase communication and social interaction skills in autistic children^1^, ^13^ and reduce their overall symptom severity.^5^ In this context, caregivers are parents in the home and LSAs in education. PACT-G includes additional strategies for integration of PACT techniques into daily routines and play at home^14^ and in education settings.^18^ PACT-G therapy, in common with the original PACT therapy, takes a staged approach based on theoretically informed precursor skills for typical social communication development^19^ and addressing atypical autistic development. The starting point is personalised and progression through the stages is individually paced according to both the child's developmental level and the adult's progress in adapting their communication with the child. Therefore, there is no expectation that all stages are completed during the therapy, and good progress can occur without completion of all stages (stages are detailed in the appendix [pp 3–4]). PACT-G differs from the original PACT in several ways. First, parents were offered 12 intervention sessions over 6 months at home, rather than 18 clinic-based sessions over 12 months, as in PACT. This reduction was for efficiency, given the demands of the multicomponent intervention design, and because good initial treatment effects had been measured after 6 months in the original PACT trial.^8^, ^12^ Second, half of these sessions were planned to use remote delivery to improve efficiency and feasibility (eg, to reduce travel time for both families and therapists). Third, a further 12 sessions over 6 months, again with 50% remote delivery, were offered in the child's education setting with their LSA, to be integrated with any other communication strategies used in that setting. Fourth, up to six home-school conversation^20^ sessions were offered to both parent and LSA together. This technique, developed to help parent–teacher communication, was intended to support the multicomponent intervention by helping parent and LSA to share experiences and promote more consistent use of strategies across settings. The intervention delivery sequence is detailed in the appendix (pp 2–4,31). From video footage of in-person and remote sessions, researchers assessed whether sessions were acceptable for inclusion in analyses using a set of prespecified criteria (appendix p 18).

Treatment as usual included any health support or intervention from education or local community services, including generic and named therapies.

Assessments were administered on entry (baseline) to the trial, at the 7-month midpoint, and at the 12-month endpoint. Demographic, clinical and family language information was collected at baseline. Three other baseline-only subtests assessments were done: the Mullen Scales of Early Learning^21^ or British Ability Scales^22^ (according to age) gave a developmental level of non-verbal abilities; Social Communication Questionnaire (SCQ) Lifetime Version^16^ gave a parent report of autism characteristics over the child's development; and the Early Sociocognitive Battery (ESB)^19^ is a primarily non-verbal clinical assessment tool suitable for use with children aged 2–5 years from diverse language backgrounds.

Adverse events were ascertained by researchers for all families included in the trial at each contact and by therapists in the PACT-G group. Adverse events included those on child health, wellbeing and behaviour, problems in education, and family events such as separation or substantial parental ill health. Adverse events resulting in death, that were life-threatening, required hospitalisation, or that caused persistent or clinically significant disability were classified as severe adverse events.^12^ All data related to adverse events and severe adverse events were reviewed blindly by principal investigators (JG, TC, HMcC, ALC, JP, VS, CA, VG, and PH), and in unblinded form by the Data Monitoring and Ethics Committee.

### Outcomes

Our primary measure was ADOS-2, as measured by masked researchers.^15^ We chose this autism diagnostic symptom measure because we could rate it in a masked manner and because of its good external validity to long-term outcomes in autism development. Developmentally-staged ADOS-2 modules are available and were selected appropriately for each child at baseline (module 1, early development non-verbal; module 1, early words; module 2, phrase speech), with the same module administered at the 12-month endpoint. Current metrics combine ADOS-2 social communication and repetitive behaviour symptom domains into a unitary total symptom score and a Calibrated Severity Score (CSS) across modules. Previous studies^9^, ^23^ have shown ADOS-2 sensitivity to treatment effects, and our previous modelling^8^ suggested that a 4-point Autism Diagnostic Observation Schedule-Generic (ADOS-G) total score change at age 3 years equates to a 20% increase in functional Vineland Adaptive Behavior Scores.^24^ Measured at baseline and at the 12 month endpoint, endpoint ADOS-2 was cross-coded across trial sites by researchers who were masked to treatment allocation. ADOS-2 administration researchers were trained to research competency (80%) and continuously assessed thereafter during regular local site and cross-site consensus meetings every 3–4 months. Inter-rater reliability was assessed on a random sample of 24 ADOS-2 videos (16 from module 1, eight from module 2) balanced across child age and treatment group. Triple coding gave intra-class correlation coefficients (ICCs) of 0·80 (95% CI 0·61–0·91) for module 1 and 0·70 (0·38–0·90) for module 2; overall 0·78 (0·62–0·88).

Secondary outcomes were split into two categories: blinded and unblinded assessments. Blinded Secondary outcomes that were assessed by investigators who were masked to treatment allocation were as follows. Researcher BOSCC^25^ is a researcher coding of autism symptoms from videotaped child–researcher interaction (baseline and 12-month endpoint), using the same autism symptom constructs as ADOS-2 but designed to detect clinically meaningful symptom change in treatment studies. Different BOSCC modules were applied according to minimally verbal versus verbal abilities. Parent and LSA BOSCC were coded from video footage of child–parent play at home (at baseline, 7 months, and 12 months) and child–LSA interaction in education (at baseline, 7 months, and 12 months) as a measure of intervention effect in the naturalistic settings in which the intervention took place. BOSCC ratings were made from the same video-capture as DCMA (described later), with administration thereby altered slightly in timing and content from that recommended (appendix pp 5–6). BOSCC administration fidelity was checked in the same fashion as for ADOS-2 throughout the trial. Formal reliability coding on 63 module 1 and 45 module 2 BOSCCs gave an ICC of 0·87 (95% CI 0·81–0·91) for module 1, 0·86 (0·76–0·92) for module 2 and overall 0·86 (0·76–0·92). Parent and LSA DCMA^8^ codes dyadic interaction for the proportion of adult communications that are synchronous and the proportion of child communications that are social initiations, avoiding non-independence within dyadic coding (appendix p 6). This measure showed mediation effects in clinic-based PACT.^11^ Formal reliability testing used double coding in varied pairings, stratified in proportion to the number of allocated videos: ICCs were 0·75 (95% CI 0·53–0·89) for parent proportions and 0·78 (0·57–0·90) for child proportions. MacArthur-Bates Communicative Development Inventories (Word and Gestures; Sentences and Grammar),^26^ Receptive and Expressive One-word Picture Vocabulary Test,^27^ and Pre-school Language Scale-5^28^ (assessed at the 12-month endpoint) were used together to measure a child's overall language level to supplement the measures of autism-specific communication included in the BOSCC and ADOS-2.

Secondary outcomes that were assessed without masking of investigators were as follows. The Developmental Behaviour Checklist – Parent (2nd edition; DBC-P):^29^ a 96-item instrument used for the assessment of behavioural and emotional problems in children and adolescents aged 4–18 years with developmental and intellectual disabilities. It includes two subscales: the disruptive and anti-social subscale and the anxiety subscale (total 36 items), which were completed by a parent or carer at the 12-month endpoint only. The Repetitive Behaviours Questionnaire (RBQ)^30^ is a 26-item parent questionnaire for assessing repetitive behaviours in autistic children. Two subscales of the RBQ were used: the RBQ insistence on sameness and sensory-motor subscales. Vineland Adaptive Behavior Scales (VABS), parent and teacher versions (P-VABS and T-VABS),^24^ include domains of communication, daily living skills, and socialisation. These scales have been used in numerous autism studies and measure child functional ability in the home and education settings. Strengths and Difficulties Questionnaire (SDQ),^31^ with parent and teacher versions, is a 25-item brief measure of psychological wellbeing in children aged 2–17 years completed by parents and teachers. Warwick-Edinburgh Mental-Wellbeing Scale^32^ is a self-rated parental wellbeing questionnaire recommended by the UK Department of Health and Social Care as the preferred measure of mental wellbeing to incorporate in studies of this kind. Child Health Utility 9D (CHU9D)^33^ is a paediatric measure of health-related quality of life comprising nine items, rated on five levels (ranging from no problems to severe problems), and designed to be completed by children aged 7–17 years. Proxy completion by parents on behalf of their child is possible for younger or developmentally disabled children. The test of parental self-efficacy (TOPSE)^34^ is a 48-item, self-report measure of parenting competence that assesses parents’ confidence in their ability to make a difference to their child's development; completed at baseline and the 12-month endpoint assessments. Child and Adolescent Service Use Schedule (CA-SUS), which includes School Service Use Schedule,^35^ were developed in the context of previous trials^8^, ^9^ to record use of therapies and service during the study.

The Working Alliance Inventory measurement,^36^ which did not form part of the prespecified analysis for this study, will be reported elsewhere. The protocol-specified outcome measure of Family History Interview (FHI) was not collected due to budgetary issues.

In additional to the efficacy outcomes, a pre-planned mechanism study was designed to exploit the ability of random allocation experiments to establish causal dependencies; in this case to investigate the poorly understood processes of generalisation of acquired skill in autistic development by collecting measures across trial contexts and investigating causal pathways of generalisation of treatments effects over time and across contexts using mediation analysis.^12^

### Statistical analysis

The PACT trial showed an effect of size 0·24 (95% CI 0·59 to –0·11) on endpoint social communication outcome (ADOS-2).^8^ The intervention strategies in PACT-G aimed to increase this effect. Using the sampsi command in Stata (version 16), for analysis of covariance with alpha of 0·05 (two-tailed), with pre-measures and post-measures correlated at 0·67 (from PACT trial), 110 participants followed-up in each group (70 preschool [aged 2 years to 4 years and 11 months] and 40 school-age [aged 5–11 years]) gave 80% power for an effect size of 0·28 and 90% power for an effect size of 0·33. Allowing for 10% attrition (compared with 4% in PACT), we proposed to recruit 244 families (rounding up to 82 per site, 52 preschool-age and 30 school-age children).^12^ No interim analyses were planned.

The statistical analysis plan (SAP) was signed-off by the Data Monitoring Committee and the Trial Steering Committee on Nov 4, 2019, before database lock and beginning analysis. Evolution of the SAP, particularly in relation to primary outcome selection, is detailed in the appendix (p 8). All analyses reported here were prespecified, with the exception of testing for variation by site (reviewer requested) in the primary treatment effect.

We tested the between-group difference in primary outcome ADOS-2 total score with an intention-to-treat (ITT) analysis using linear regression, stratified by ADOS-2 module, covarying by baseline ADOS-2 total and dummy variables for site, gender, and age group (preschool age *vs* school age). Baseline data were almost always complete and drop-out rates were very low. We estimated models using maximum likelihood under an assumption of missing at random. We applied standard residual diagnostics and adopted skew-minimising transformations where required, but this was not found to be necessary. We calculated an overall effect size by pooling stratum-specific estimates for strata defined by the ADOS-2 module, inversely weighted by their precision, using a 95% CI estimated from 5000 bootstrap replicates. We did site and age-group specific analyses of ADOS2 as primary outcome.

Analysis of all secondary measures of outcome included covariate adjustment for site, gender, and age group (preschool *vs* school age). We stratified the repeated parent, teacher, and researcher BOSCC analyses by module, in multiple group structural equation modelling using the method(mlmv) full-information maximum likelihood estimator. We assessed the repeated assessments as correlated regressions with post-baseline assessment equations also including treatment allocation with a coefficient common across module. The repeated DCMA assessments of parent synchrony and child initiation were also analysed similarly, but using single-group SEM. The endpoint Child Health Utility 9-D was analysed using regression, covarying for baseline CHU-9D score. We analysed our other secondary outcomes as composite outcomes. Details of the construction of the composites and the associated analyses for treatment effects are given in the appendix (p 7).

The complier-average causal effect (CACE) estimator used a similar model to that for the primary analysis, but replaced treatment allocation by a measure of the total number of PACT-G treatment sessions received by each child that met the therapists criterion for skill acquisition (and set to zero in the treatment-as-usual group). Assuming linearity, we extracted an estimate for the effect for eight sessions from the model. Both the count of the acceptable sessions and the criterion of eight sessions were prespecified.

In our mechanism analysis, we used linear structural equation models to test for mediation of the intervention on primary and secondary symptom outcomes (ADOS-2, researcher BOSCC) through DCMA and BOSCC at home and in education settings, and to examine multiple pathways through DCMA and BOSCC at home and in education settings to the researcher-assessed symptom outcomes. We used bootstrapping to produce valid SEs for the indirect effects. We adjusted all analyses for baseline measures of BOSCC and DCMA, primary outcome (ADOS-2), and putative measured confounders. We built on our previous methodological and applied work in this context^37^ to include repeated measurement of mediators and outcomes to account for measurement error and baseline confounding.

We did all analyses using Stata (version 17.0). This study is registered with the ISRCTN registry, ISRCTN 25378536

### Role of the funding source

The funder of this study guided the primary outcome choice but had no other role in study design, data collection, data analysis, data interpretation, or writing of the report.

## Results

**Figure 1:**
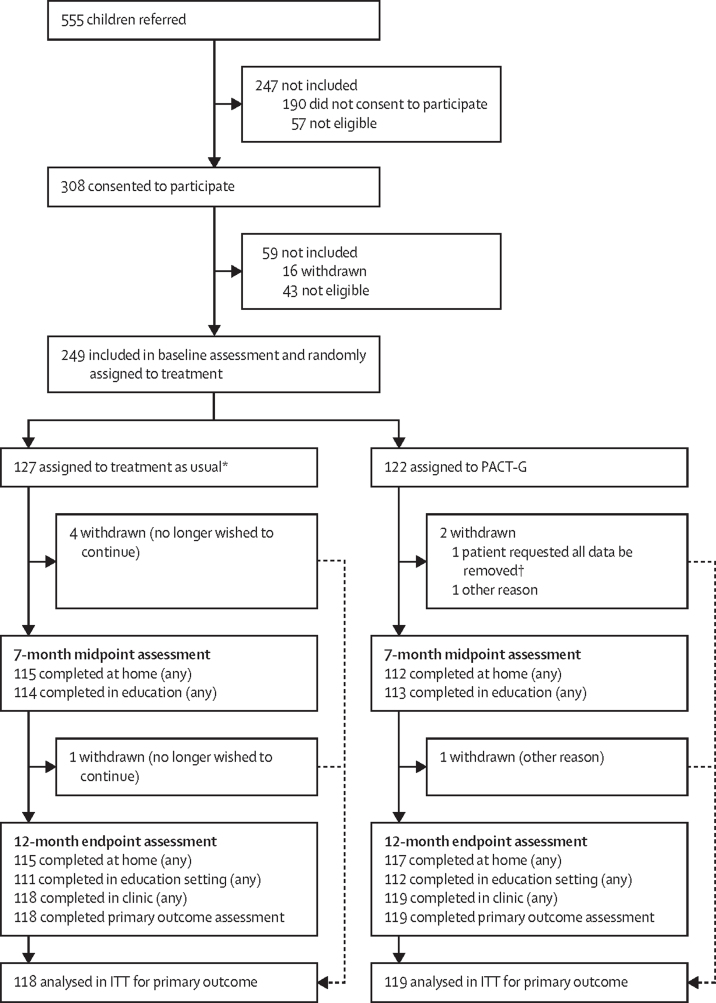
Trial profile ITT=intention-to-treat. PACT-G=Paediatric Autism Communication Trial-Generalised. *One family inadvertently received PACT-G treatment. †Was not included in ITT analysis.

Between Jan 18, 2017, and April 19, 2018, 555 children were referred, of whom 249 were eligible, agreed to participate, and were randomly assigned to either PACT-G (n=122) or treatment as usual (n=127; figure 1; details of drop-outs and attrition are shown in the appendix [p 11]). One participant family withdrew after assignment to PACT-G and requested their data be removed from the study, hence they were not included in the intention-to-treat population. Baseline characteristics for the ITT population are shown in table 1. Participant children were ethnically diverse but predominantly White (149 [60%] of 248), male (197 [79%]), and with only English spoken at home (197 [79%]). There was substantial baseline autism severity, consistent with the inclusion criteria, balanced across groups. With few exceptions, children in the older age group scored 2SD or less below the population mean for the Mullen Developmental Quotient—a lower development quotient than the younger age group, consistent with inclusion criteria (appendix pp 11).

**Table 1:** Baseline demographic, verbal IQ, and Early Sociocognitive Battery characteristics, intention-to-treat population

		**Treatment as usual (n=127)**	**PACT-G (n=121)**
**Age**			
Preschool-aged		77 (61%)	74 (61%)
Mean, years		3·9 (0·7)	4·1 (0·6)
School-aged		50 (39%)	47 (39%)
Mean, years		6·9 (1·4)	7·4 (1·6)
**Gender**			
Female		27 (21%)	24 (20%)
Male		100 (79%)	97 (80%)
**Ethnicity**			
White		73 (57%)	76 (63%)
Black		21 (17%)	19 (16%)
Asian		16 (13%)	13 (11%)
Mixed		17 (13%)	6 (5%)
Other		0	7 (6%)
**Second parent in household**			
No		28 (22%)	27 (22%)
Yes		99 (78%)	94 (78%)
**Languages spoken to child at home**			
English only		98 (77%)	99 (82%)
Other only		3 (2%)	1 (1%)
English and other		26 (20%)	21 (17%)
**Non-verbal IQ**			
Mullen visual standard t		25·2 (11·7)	22·9 (8·0)
Mullen visual raw score		28·3 (7·8)	28·5 (7·0)
Mullen fine motor standard t		22·8 (8·6)	21·8 (7·3)
Mullen fine motor raw score		26·3 (6·9)	27·2 (6·5)
Early Sociocognitive Battery		124 (98%)	116 (96%)
	Social response	2·6 (3·4)	2·7 (3·2)
	Joint attention	6·3 (5·4)	6·1 (5·2)
	Symbolic total	4·1 (5·0)	3·8 (4·2)

Data are n (%) or mean (SD). Mullen=Mullens Scales of Early Learning.

PACT-G was implemented in homes with a median of 10 (IQR 8–12) of 12 sessions per participant; 765 [64%] of 1189 sessions were in-person, 424 [36%] were remote). Study therapists judged 45 (6%) of 765 in-person sessions and 60 (14%) of 424 remote sessions unacceptable; remote sessions were usually determined to be unacceptable because of technical difficulties or absence of reliable video-sharing equipment. In education, a median of 8 (IQR 5–10) of 12 PACT-G sessions were delivered (632 [66%] of 952 were in-person, and 320 [34%] were remote), with 41 (6%) of 632 in-person and 51 (16%) of 320 remote sessions considered unacceptable by therapists. By end of treatment, the home-based therapy had generally reached a more advanced stage in the treatment manual than the education-based therapy (appendix p 12). Most PACT-G therapy partners (92 [79%] of 117) in education were LSAs (appendix p 14). Of 117 children who engaged in PACT-G therapy in education, 40 (34%) had a change in the education professional delivering PACT-G between baseline and the 7-month midpoint, 44 (38%) had this change between the 7-month midpoint and the 12-month endpoint, and 22 (19%) at both timepoints. Three participants in the intervention group received fewer than eight intervention sessions out of a possible 24 (ie, below the minimum acceptable number). In the educational setting, the number of sessions LSAs had that were acceptable ranged from none to 12 with 14 participants having fewer than four acceptable sessions, and in the home setting the range was two to 12, with six participants receiving fewer than four acceptable sessions. Manual fidelity was not rated for remote sessions but was independently assessed in 43 (3·1%) of 1397 in-person sessions, randomly balanced across delivery context, trial timepoint, and therapist. Prespecified fidelity criteria (appendix pp 43–46) were met in 37 (86%) of 43 sessions. A mean of 2·2 (SD 1·5, from N=75) home-school conversation sessions per participant were delivered, compared with a prespecified minimum acceptable threshold of 3 sessions per participant. Treatment as usual received was balanced across treatment groups, with most parents reporting unnamed or eclectic communication-focused therapy, and a small number of named therapies (appendix pp 14–15). One family assigned to treatment as usual inadvertently received PACT-G treatment; their data were reported and analysed as part of the treatment as usual group.

**Figure 2:**
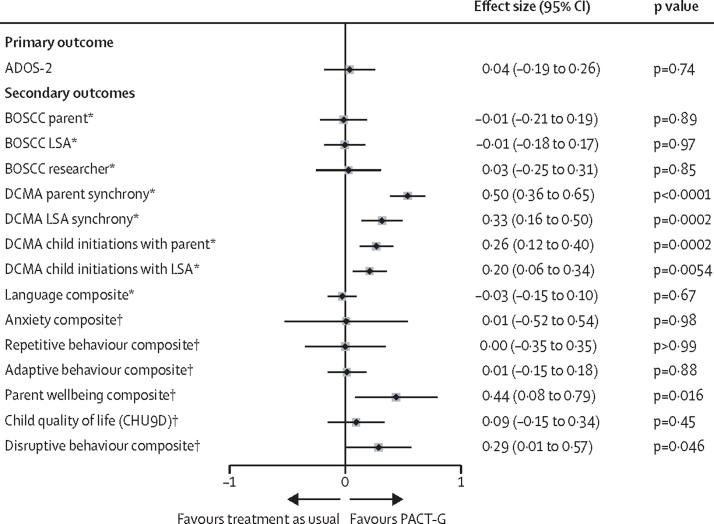
Outcome measure effect sizes ADOS-2=Autism Diagnostic Observation Schedule-second edition. BOSCC=Brief Observation of Social Communication Change. CHU9D=Child Health Utility 9D. DCMA=Dyadic Communication Measure for Autism. LSA=learning support assistant. PACT-G=Paediatric Autism Communication Trial-Generalised. *Assessed in a masked fashion. †Assessed in an unmasked fashion.

For the primary analysis, the median follow-up was 363 days (IQR 356–377). 12-month endpoint ITT analysis of ADOS-2 primary outcome (n=118 in treatment as usual group and n=119 in PACT-G group; table 2; figure 2) estimated an adjusted mean difference of 0·04 (95% CI –0·19 to 0·26; p=0·74). We found no difference in treatment effect by age group, based on the bootstrap p value of the pooled stratum-specific estimates of the treatment difference; or by site (appendix p 18). Mapping this difference onto the ADOS-2 calibrated severity score showed little substantive between-group difference (appendix p 9) but effects showed some variation by ADOS-2 module (appendix p 17). Pre-planned complier average causal effect (CACE) analysis estimated the treatment effect in those who received above the prespecified minimum threshold of eight sessions. The across-strata pooled CACE estimate was not significant (0·02; p=0·76) and assuming a linear dose–response association, 112 sessions would have been required to have a clinically significant effect size of 0·28.

**Table 2:** Primary and secondary outcomes

			**Baseline**		**12-month endpoint**	
			Treatment as usual (n=127)	PACT-G (n=121)	Treatment as usual (n=127)	PACT-G (n=121)
**Primary outcome**						
ADOS-2 total score			127	121	118	119
	Module 1: non-verbal		21·4 (2·9)	20·9 (2·5)	20·9 (3·2)	20·4 (3·4)
	Module 1: verbal		17·6 (3·1)	17·3 (2·6)	16·8 (4·6)	16·0 (4·2)
	Module 2: young		15·2 (2·8)	15·5 (4·0)	11·5 (4·3)	13·7 (3·6)
	Module 2: old		15·0 (4·4)	17·7 (3·5)	15·5 (3·2)	17·3 (3·5)
	Total score		18·6 (4·0)	18·8 (3·6)	17·5 (5·0)	17·6 (4·5)
ADOS-2 social-affect			127	121	118	119
	Module 1: non-verbal		16·4 (2·7)	16·1 (2·1)	16·4 (2·5)	15·7 (2·5)
	Module 1: verbal		13·9 (2·6)	13·3 (2·5)	12·8 (3·7)	12·6 (3·2)
	Module 2: young		12·4 (3·0)	12·1 (2·9)	10·1 (4·2)	11·3 (3·1)
	Module 2: old		11·8 (3·5)	13·8 (2·5)	11·8 (2·8)	13·7 (2·6)
	Subscale score		14·5 (3·3)	14·5 (2·8)	13·8 (4·0)	13·8 (3·3)
ADOS-2 restrictive and repetitive behaviour			127	121	118	119
	Module 1: non-verbal		5·0 (1·5)	4·8 (1·6)	4·5 (1·6)	4·8 (1·5)
	Module 1: verbal		3·7 (1·5)	3·9 (1·5)	3·9 (1·8)	3·3 (1·7)
	Module 2: young		2·8 (1·2)	3·4 (1·4)	1·4 (0·9)	2·4 (1·5)
	Module 2: old		3·2 (1·7)	3·9 (1·4)	3·8 (1·6)	3·7 (1·9)
	Subscale score		4·1 (1·7)	4·3 (1·6)	3·8 (1·9)	3·8 (1·8)
**Secondary outcomes**						
Language composite						
	MacArthur CDI		120	119	101	107
		Words understood	202·4 (128·4)	213·4 (134·7)	260·6 (130·6)	264·8 (121·9)
		Words understood and said	139·1 (141·5)	152·1 (144·3)	188·7 (156·1)	175·7 (159·3)
	Language scores		127	120	118	116
		Receptive one-word n at basal	57	46	42	39
		Receptive one-word	29·4 (22·5)	32·1 (19·3)	42·2 (21·9)	43·2 (20·0)
		Expressive one-word n at basal	62	58	52	49
		Expressive one-word	28·2 (19·2)	32·0 (12·9)	42·0 (21·7)	42·5 (18·5)
	Pre-school language scale					
		Receptive	..	..	102	92
		Subscale score	..	..	59·5 (18·9)	57·7 (13·7)
		Expressive	..	..	102	92
		Subscale score	..	..	59·3 (20·0)	57·5 (14·9)
Anxiety composite						
	SDQ					
		Teacher emotional	123	113	104	104
		Subscale score	2·1 (1·9)	2·2 (2·1)	2·5 (1·9)	2·2 (1·9)
		Parent emotional	118	118	102	107
		Subscale score	3·1 (2·1)	2·5 (2·2)	3·0 (2·2)	2·6 (2·1)
	DBC-P					
		Anxiety	..	..	42	38
		Subscale score	..	..	5·3 (4·1)	6·2 (4·1)
Repetitive behaviour composite						
	SCQ					
		Parent repetitive	126	121	..	..
		Subscale score	6·5 (1·8)	6·4 (1·7)	..	..
	RBQ					
		Insistence on sameness	119	116	100	105
		Subscale score	8·6 (6·4)	8·3 (5·9)	8·6 (6·2)	7·8 (5·6)
		Sensory motor	119	116	100	105
		Subscale score	10·9 (5·3)	9·4 (4·7)	10·2 (5·4)	9·1 (5·3)
Adaptive behaviour composite						
	SDQ					
		Teacher prosocial	123	112	104	105
		Subscale score	1·6 (1·9)	1·8 (2·0)	2·2 (2·5)	2·5 (2·3)
		Parent prosocial	118	118	102	107
		Subscale score	2·4 (2·1)	2·6 (2·2)	3·2 (2·4)	3·5 (2·3)
	Vineland adaptive behaviour					
		Parent	127	120	111	117
		Total score	62·2 (10·3)	61·6 (8·9)	63·9 (12·8)	62·7 (11·5)
		Teacher	117	110	103	105
		Total score	58·9 (15·7)	57·4 (14·1)	58·1 (16·5)	58·9 (16·5)
Parental wellbeing composite						
	WEMWBS		117	117	103	104
		Total score	49·6 (9·0)	48·9 (9·9)	48·8 (9·5)	50·6 (9·9)
	Parental self-efficacy		120	113	99	106
		Total score	368·7 (57·2)	368·5 (53·5)	370·5 (55·5)	379·8 (53·1)
Child health-related quality of life						
	CHU9D		116	114	103	104
		Total score	17·4 (5·4)	16·7 (5·3)	16·4 (5·3)	15·5 (4·6)
Disruptive behaviour composite						
	DBC					
		Disruptive/antisocial	..	..	42	38
		Subscale score	..	..	12·9 (9·8)	14·7 (9·2)
	SDQ					
	Externalising—parent		118	118	102	107
		Subscale score	2·9 (1·5)	2·8 (1·9)	3·0 (1·7)	2·4 (1·5)

Data are n or mean (SD), unless otherwise indicated. ADOS-2=Autism Diagnostic Observation Schedule-second edition. CHU9D=Child Health Utility 9D. DBC=Disruptive Behaviour Questionnaire. DBC-P= DBC=Disruptive Behaviour Questionnaire-parent. MacArthur CDI=MacArthur-Bates Communicative Development Inventories. PLS=Preschool Language Scale. RBQ=Repetitive Behaviours Questionnaire. SCQ=Social Communication Questionnaire. SDQ=Strength and Difficulties Questionnaire. WEMWBS=Warwick-Edinburgh Mental Wellbeing Scale.

Effect estimates for secondary outcome measures and composites (figure 2, Table 2, Table 3) showed no significant treatment effects on autism symptom behaviours on the BOSCC in home (–0·01 [95% CI –0·21 to 0·19; p=0·89]), in education (–0·01 [–0·18 to 0·17; p=0·97]), or research (0·03 [–0·25 to 0·31]; p=0·85]) settings. Significant treatment effects were seen on the proximal intervention target of parent synchronous responses to the child in home (DCMA: effect size 0·50 [95% CI 0·36 to 0·65; p<0·0001]) and LSA synchronous response to child in education (0·33 [0·16 to 0·50; p=0·0002]), and on child dyadic social communication initiations with parent (0·26 [0·12 to 0·40; p=0·0002]) and LSA (0·20 [0·06 to 0·34; p=0·0054]). No significant treatment effects were seen on parent reported language, anxiety, repetitive and adaptive behaviour, or child health-related; however, significant effects were seen in parental self-reported wellbeing and in the combined parent and teacher report of child disruptive behaviour severity (table 2, figure 2).

**Table 3:** BOSCC and DCMA interaction-based assessments

	**Baseline**			**7-month midpoint**			**12-month endpoint**		
	Treatment as usual (n=127)	PACT-G (n=121)	Overall (n=248)	Treatment as usual (n=127)	PACT-G (n=121)	Overall (n=248)	Treatment as usual (n=127)	PACT-G (n=121)	Overall (n=248)
**BOSCC**									
Researcher N	124	114	238	..	..	..	118	115	233
Researcher	39·9 (9·3)	40·0 (8·3)	40·0 (8·8)	..	..	..	37·0 (10·9)	37·0 (11·2)	37·0 (11·0)
LSA N	119	118	237	114	111	225	111	112	223
LSA	37·8 (9·8)	38·7 (10·0)	38·2 (9·9)	35·2 (10·1)	36·8 (11·0)	36·0 (10·6)	35·1 (11·5)	36·4 (11·6)	35·7 (11·5)
Parent N	124	112	236	109	112	221	112	115	227
Parent	36·1 (9·4)	36·7 (8·7)	36·4 (9·1)	33·5 (10·1)	34·9 (10·6)	34·2 (10·4)	33·7 (9·8)	34·4 (11·3)	34·0 (10·6)
**DCMA-LSA**									
LSA N	125	119	244	109	113	222	110	114	224
LSA synchronous response	0·32 (0·14)	0·31 (0·13)	0·31 (0·13)	0·34 (0·12)	0·46 (0·18)	0·40 (0·16)	0·32 (0·12)	0·44 (0·17)	0·38 (0·16)
Child N	125	118	243	109	112	221	109	113	222
Child initiations	0·26 (0·17)	0·24 (0·19)	0·25 (0·18)	0·24 (0·18)	0·32 (0·22)	0·28 (0·20)	0·25 (0·18)	0·32 (0·21)	0·29 (0·20)
**DCMA-parent**									
Parent N	124	121	245	115	112	227	115	115	230
Parent synchronous response	0·35 (0·11)	0·33 (0·13)	0·34 (0·12)	0·37 (0·14)	0·47 (0·18)	0·42 (0·17)	0·36 (0·13)	0·43 (0·18)	0·40 (0·16)
Child N	124	121	245	114	110	224	113	115	228
Child initiations	0·29 (0·21)	0·26 (0·20)	0·28 (0·20)	0·25 (0·21)	0·36 (0·25)	0·30 (0·24)	0·24 (0·20)	0·29 (0·22)	0·27 (0·21)

Data are n or mean (SD). BOSCC=Brief Observation of Social Communication Change. DCMA=Dyadic Communication Measure for Autism. LSA=learning support assistant.

In the mechanism analysis (appendix pp 10, 17), treatment effects on improved child dyadic social initiation observed in home were mediated by improvements in parent dyadic synchronous response with the child (indirect effect 0·05 [SE 0·01; 95% CI 0·03 to 0·08; p<0·0001]) and treatment effects on child dyadic social communication observed in education were mediated by improvements in LSA dyadic synchronous response with the child (0·01 [SE 0·01; 95% CI 0·01 to 0·06]; p=0·0028]; appendix p 17). Additionally, improved child dyadic initiation showed mediation of a (non-significant) endpoint BOSCC at home (indirect effect –0·78 [SE 0·34; 95% CI –1·44 to –0·11; p=0·022]), although not in education (indirect effect –0·67 [SE 0·37; 95% CI –1·40 to 0·06; p=0·073]) settings (appendix p 17).

There were 46 adverse events, of which six were classified as serious (table 4). Seven events judged possibly related to the study had a similar incidence across treatment groups. One serious adverse event judged possibly related to the study involved impact on parental mental health of the autism diagnostic process and collaborative nature of the PACT-G therapy.

**Table 4:** Adverse events, by gender

		**Treatment as usual**		**PACT-G**		**Overall**	
		Female (n=27)	Male (n=100)	Female (n=24)	Male (n=97)	Female (n=51)	Male (n=197)
Had at least one adverse event		7 (26%)	15 (15%)	5 (21%)	19 (20%)	12 (24%)	24 (12%)
Serious adverse event requiring hospitalisation		0	2/15 (13%)	1/5 (20%)	1/19 (5%)	1/12 (8%)	3/34 (9%)
Serious adverse event resulting in persistent or clinically significant disability		0	0	1/5 (20%)	1/19 (5%)	1/12 (8%)	1/34 (3%)
Type of adverse event (people)							
	Reduction in school attendance	0	1 (1)	0	0	0	1 (1)
	Other issues with school	1 (1)	0	0	1 (1)	1 (1)	1 (1)
	Relationship breakdown (parent)	0	0	0	5 (5)	0	5 (5)
	Substantial change in child behaviour or wellbeing	4 (4)	9 (9)	3 (3)	8 (8)	7 (7)	17 (17)
	Clinically significant family illness	0	1 (1)	1 (1)	0	1 (1)	1 (1)
	Death in immediate family	0	1 (1)	0	1 (1)	0	2 (2)
	Other personal or family issue	2 (2)	2 (2)	1 (1)	2 (2)	3 (3)	4 (4)
	Other	0	1 (1)	0	2 (2)	0	3 (3)
Relationship to study treatment							
	Possibly related	3/7 (43%)	1/15 (7%)	2/5 (40%)	1/19 (5%)	5/12 (42%)	2/34 (6%)
	Not related	4/7 (57%)	14/15 (93%)	3/5 (60%)	18/19 (95%)	7/12 (58%)	32/34 (94%)

Data are n (%), n/N (%) or number of events (number of people).

## Discussion

We found that, compared with treatment as usual, treatment with PACT-G had no effect on the blinded primary outcome of autism symptoms, secondary outcome of symptom change in treatment or research settings, parent report of language or parent or teacher reported adaptive behaviour outcomes. Treatment with PACT-G did improve dyadic social communication between child and parent at home and between child and LSA in education. Additionally, we found two treatment effects of importance to families and education settings: improvement in parental wellbeing, known to be a salient factor for families with an autistic child, and significantly reduced overall child disruptive behaviour on combined LSA and parent report. The clinic-based PACT intervention had produced similar improvements in parent confidence and family and child function.^38^

Our findings on autism symptom outcome differ from those of studies of the original clinic-delivered PACT with parents alone, for which effects were found on social communication symptom outcomes in one trial^7^ but not another,^8^ as well as improving the full symptom phenotype, sustained over treatment and the 6-year follow-up period.^9^ The findings on child–parent social communication outcomes of PACT-G replicate the positive effects found in the previous trials, but notably at about half the effect size (PACT-G effect size: parent synchrony 0·54, child initiation 0·27; PACT effect size:^8^ parent synchrony 1·22, child initiations 0·41). The PACT-G findings are more in keeping with recent systematic^1^, ^3^, ^4^ and narrative^2^, ^5^ reviews that autism interventions frequently affect proximal targets but more rarely affect change in a standard measure of autism symptoms across contexts, people, and over time. Within the education setting, there are few publications on autism interventions in education, and they have found no effect on formally assessed autism symptom outcomes. Two comprehensive curriculum-embedded interventions^39^, ^40^ run over a school year, one of which had at last 10 h per week of additional one-to-one instruction, have shown improvements in observed classroom social functioning and academic performance. However, briefer discrete external health interventions delivered within the classroom^41^ have found a similar pattern of results to PACT-G, with treatment effects on proximal outcomes not accompanied by distal effect. The reduction in levels of child disruptive behaviour across home and education settings seen with PACT-G is highly salient, because emotional and behavioural difficulties are common in autistic children, have a significant effect on child and family wellbeing, and restrict family life opportunities; therefore, they need addressing alongside social communication development.^42^ Our findings that PACT-G, as a briefer multicomponent intervention across home and education, did not produce greater effects on outcome than the clinic-based version (PACT) is consistent with a meta-analysis of 213 universal multicomponent interventions for social and emotional skills development across community and education,^43^ which found no benefit of multicomponent over single-component programmes. The authors of this meta-analysis speculate that multicomponent interventions are more challenging to implement and less likely to adequately deliver their constituent crucial components for effect than are single component interventions.

Our trial had several limitations. The intervention dosage and adherence differed from the PACT trial; sessions were spread across home and education, with 12 sessions offered specifically to parents compared with 18 in PACT, and about a third of sessions delivered remotely rather than in person. Moreover, treatment was often not implemented as intended; on average ten (83%) of 12 sessions in home and eight (67%) of 12 in education were attended, compared with 16 (89%) of 18 in clinic-delivered PACT.^8^ Remote sessions were more likely to be rated inadequate by therapists than in-person sessions and independent fidelity rating of remote sessions was not possible for technical reasons. The home-school conversation sessions were not delivered as planned, with each caregiver pair per child on average receiving fewer than the minimum acceptable threshold, which was largely because of time availability of the therapist. The therapy partners in education changed frequently and a minority who were not LSAs might have had less overall involvement with the child. This change in education therapy partner affected intervention delivery for the children, but also the baseline and 12-month endpoint BOSCC and DCMA research assessments in the educational setting, because these measures depend on consistent dyadic participants between assessment points. Gender-specific analysis of the efficacy endpoints could not be done because the numbers were too small. Finally, we do not yet have developmental follow-up data; in which other similar intervention studies^9^, ^23^, ^44^, ^45^ have found emerging symptom efficacy effects after intervention endpoint.

The mechanism study used approximately 3500 inde-pendently blind-coded interaction videotapes across context and time, an assessment we believe is unprecedented in scale for a developmental study. The analysis showed that improvement in adult synchronous response resulted in increased child dyadic social communication in both home and education delivery contexts; a replication of the initial mediation step in the clinic-based PACT trial.^11^ Because there was no treatment effect on outcome symptoms, we could not test for the second, within-child, generalisation step found in the PACT trial (improved child dyadic communication strongly mediating reduced severity of independently rated child autism outcome symptoms).^11^ However, the significant mediation between dyadic child communication change in home and outcome BOSCC can be considered some evidence for this latter pathway, albeit not seen in ADOS-2. A possible reason for the absence of second-step transmission in this trial is the reduced effect size on caregiver–child dyadic interaction, which, with an effect size approximately half that seen in the original PACT trial,^8^, ^11^ was insufficient to drive the full second stage path to symptom change. If so, a potential reason for this reduced first stage effect is session dose. With parents, a mean of ten in-person or remote sessions were delivered in PACT-G compared with 16 in-person in PACT. Previously reported classroom-embedded interventions that have achieved objective educational and social interaction gains were delivered daily throughout a school year;^39^, ^40^ a substantially higher dose equivalent than PACT-G. Insufficient dose was considered possible in the classroom-based implementation of a related social communication therapy,^41^ which also found only positive proximal effects. Results of our CACE analysis do not support a simple sessional dose explanation for the PACT-G findings: however, the analysis is based on assumptions of linear dose-response relationship, whereas non-linear dose threshold effects are commonly found.^46^ Session quality was potentially compromised by remote delivery and the separate difficulties outlined in home and education settings, along with fewer than planned home-school conversations. These difficulties were most commonly related to the environmental conditions rather than therapist training or quality. The difference in PACT-G therapy stages reached between home and education settings might have added complexity for the children. Notably, all of these considerations are commonly promoted features of adapted intervention implementation across mental health, with remote delivery becoming very prevalent during the COVID-19 pandemic. Our results suggest that for autistic people caution is needed in assuming a simple transfer of efficacy from standard treatments when adapted in such ways.

A general interpretation of our findings is that the design steps taken in PACT-G to amplify the transmission of proximal child social communication gains into generalised symptom outcomes, had the unintended consequence of halving the initial effect size of the intervention on proximal adult–child dyadic interaction, and that this, in turn, reduced symptom outcome efficacy. Clinically, the trial showed that implementation of this relatively low intensity, video-aided intervention can be successfully scaled into home and education, with positive effects on dyadic communication skills, parental wellbeing, and child disruptive behaviour. However, it raises caveats around poor generalisation to symptom change compared with clinic-based PACT. We conclude that the original 18 session clinic-based PACT with parents is now indicated for clinical work, and that implementation in education should be undertaken with PACT-G's results in mind. Future research of psychosocial treatments in a rapidly changing mental health delivery environment should prioritise specific dose-finding and remote implementation studies, alongside greater understanding of the effect of delivery setting and multiple therapy components.

**This online publication has been corrected. The corrected version first appeared at thelancet.com/psychiatry on March 30, 2022**

## Data sharing

We will make data available to the scientific community with as few restrictions as feasible, while retaining exclusive use until the publication of major outputs. All data requests should be submitted to the corresponding author for consideration. Access to anonymised data might be granted following review.

## Declaration of interests

JG and AP are National Institute for Health Research (NIHR) senior investigators (NIHR NF-SI-0617-10168 for JG and NF-SI-0617-10120 for AP). RE is supported by an NIHR Research Professorship (NIHR300051) and is a member of NIHR Clinical Trials Unit Standing Advisory Committee and Health Technology Assessment Clinical Evaluation and Trials Committee. JG and CA receive director's fees from a not-for-profit PACT training company IMPACT (CiC 10902031). SC is funded by the UK Medical Research Council (MRC). TC has served as a paid consultant to F Hoffmann-La Roche and Servier, and has received royalties from Sage Publications and Guilford Publications. AP receives book and questionnaire royalties from Western Psychological Services, Oxford University Press, and Imperial College Press. All other authors declare no competing interests.
