# Correction to *Lancet Psychiatry* 2022; 9: 307–20

**DOI:** 10.1016/S2215-0366(22)00113-4

**Published:** 2022-05

*Green J, Leadbitter K, Ellis C, et al. Combined social communication therapy at home and in education for young autistic children in England (PACT-G): a parallel, single-blind, randomised controlled trial.* Lancet Psychiatry *2022; **9:** 307–20*—In the Article, Jessica Rose and Claire Bennett should have been included in the Acknowledgments. This correction has been made to the online version as of March 30, 2022.

