# Supplementary material for: Combined social communication therapy at home and in education for young autistic children in England (PACT-G): a parallel, single-blind, randomised controlled trial
Source: Lancet Psychiatry. 2022 Apr;9(4):307–20. doi: 10.1016/S2215-0366(22)00029-3 (PMC9630149; doi:10.1016/S2215-0366(22)00029-3)
Supplement: Supplementary appendix [file mmc1.pdf]

THE LANCET
Psychiatry

Supplementary appendix

This appendix formed part of the original submission and has been peer reviewed. We post it as supplied by the authors.

Supplement to: Green J, Leadbitter K, Ellis C, et al. Combined social communication therapy at home and in education for young autistic children in England (PACT-G): a parallel, single-blind, randomised controlled trial. *Lancet Psychiatry* 2022; **9:** 307–20.

COMBINED SOCIAL COMMUNICATION THERAPY IN HOME AND EDUCATION FOR YOUNG CHILDREN WITH AUTISM (PACT-G) – A PARALLEL, SINGLE-BLIND, RANDOMISED CONTROLLED TRIAL

APPENDIX

Further details on the study population

Inclusion Criteria

- Age 2–10 years
- Diagnosis of autism spectrum disorder (ASD) given by referring services.
- Meeting criteria for autism on the ADOS-2
- Scoring ≥ 15 (school-aged) and ≥ 12 (pre-school) on the Social Communication Questionnaire (SCQ-Lifetime) – the accepted clinical cut-offs for children at these ages
- Children aged 5 years and over are between P3 and P8 for the English curriculum
    - In England at the time of the study, P scales described targets for children aged 5-16 years with special educational needs. P3 communication skills indicated that a child is beginning to use 'intentional communication'. P8 was taken to represent an expressive language age-equivalent of approximately 4 years in a typically developing child. For children under 5 years, there was no selection on language criteria given the wide language heterogeneity independent of core autism symptoms. For children above 5 years, we included children who still were in early developmental stages of social communication despite their chronological age. PACT addresses social-pragmatic impairments through indirect adult-mediated strategies and support. For children with more advanced social communication and language skills alternative therapies can be delivered directly to the child aiming to increase self-refection/ awareness and direct child-mediated strategies
- Parents with sufficient English to potentially participate in the intervention and who speak English to their child at least some of the time

Exclusion Criteria

- Sibling with autism already in the trial
- Participation in the PACT-G pilot phase
- Children aged ≤ 12 months non-verbal age-equivalent level
- Epilepsy not controlled by medication
- Children with an identified genetic disorder that would impact on ability to participate or affect validity of data
- Severe hearing or visual impairment in parent or child
- Current severe learning disability in the parent, or current severe parental psychiatric disorder
- Current safeguarding concerns or other family situation that would affect child/family participation in the trial
- No agreement to participate from child's education setting

Further details on the PACT-G intervention

PACT-G retains effective elements of the original PACT manual with additional new features to aid generalisation, make modifications for older children and incorporate recent advances in research. Changes made to PACT-G from the original PACT are summarised:

- *Individual differentiation*

Modifications made for older children include more individual differentiation so that intervention begins at a point appropriate to the child's initial level of object interest and social engagement.

- *Home-School Conversation (HSC)*

Monthly HSCs provide a 'bridge' between the school and home contexts that facilitate shared understanding and exchange of information pertaining to the child's communication needs and progress with the PACT-G intervention. The monthly school based HSC sessions aim to generalise social-communication skills achieved in the 1:1 play sessions into everyday life routines, for example mealtimes, outdoor play, dressing, washing and to establish their spontaneous and independent use by the child. Parents and education staff use a diary to record generalisation and spontaneously occurring 'PACT-G moments' occurring at other times of the day. The structure of the HSC involves explore, focus, summary.

- *Play materials*

Play materials that match age, motivation and developmental level of the child are selected by the therapist at each PACT-G session. Some stages require specific consideration of the choice of toys; other stages include daily routines and interaction.

- *Selecting video clips for feedback*

Modification to PACT-G include the therapist selecting positive clips of adult demonstrated skill e.g. successful modelling and increased confidence in playing with the child, instead of parent selected video clips.

- *Combined remote Skype/Phone sessions*

Remote Skype or phone sessions occur once monthly between alternate in-person PACT-G home and school visits, i.e., a total of 10 remote sessions divided between home and school. These intervening remote sessions aim to achieve maintenance and generalisation of skills practiced in the PACT-G in-person visits.
The therapist asks the school/parent to make short video clips of interaction demonstrating the skills practiced/accomplished. The video clip may demonstrate PACT-G Stage-specific practice or more spontaneous generalisation of goals in other daily routines or settings, depending on the context. Video clips are sent securely to the therapist prior to the remote session for therapist advance review, to select clips reflecting the goals in the programme and other PACT-G skills. Where it is not possible to send the generalisation examples before the remote session, an adult-play video is made during the remote session for discussion.

- *Stage-specific modifications*

New techniques include current research modifications. Speaking for the Child is included, a technique developed in the attachment intervention Video-feedback Intervention to promote Positive Parenting and Sensitive Discipline (VIPP SD; Juffer, et al., 2008). In stage 1 the therapist encourages the parent, while watching the video, to describe or 'provide subtitles' for the signals and behaviours of the child. This is a useful technique for helping the parent, or other relevant adult to observe and interpret the child's thoughts, feelings and intentions. Following PACT mediational analysis, PACT-G stage 2 synchrony is now given greater prominence, and the threshold to progress to stage 3 is increased to 50% synchrony. Stage 5 includes a wider range of daily life generalisation opportunities.

Description of the PACT–G intervention:

Prior to starting the intervention, a school and home visit is conducted to introduce the intervention, explore the context and set expectations. 12 intervention sessions are delivered over a period of 6 months in both home and education setting, with sessions alternating between monthly face-to-face and remote Skype/phone delivered sessions. Intervention in the educational setting usually begins after the parent has commenced intervention; the start time is flexible to best fit with school terms, but a period of overlap is always included. A member of staff (usually a Learning Support Assistant; LSA) receives initial training to increase their awareness of the child's competence in key social communication skills and knowledge about the range of PACT-G interaction strategies. These sessions mirror those for the parents using video aided review of PACT-G strategies appropriate to the individual child. Implementation compliments other strategies for communication that may already be in use in the educational setting.

Each PACT-G session begins with a discussion of previous goals, progress made since the last session and amount of practice. The adult and therapist watch a 5-minute video of an adult-child play session, either a video made by the therapist during the session or an adult-made video in the home or school context. The child does not take part in the feedback section of the session, but may be present in some cirumstances depending on childcare/education setting constraints. The therapist uses guided discovery techniques to facilitate the adult to identify actions that lead to child communication and to adopt PACT-G strategies in their interaction with the child. In addition to the 1:1 play sessions, the adult's knowledge of the child, and observations of child responses in other daily contexts or routines, are used to identify situations where PACT-G principles and individual child goals may be practiced. The therapist uses record sheets to note communication responses observed during the video playback for each Stage of PACT-G. Adults are assisted to set goals for themselves, based on the interaction strategies discussed. They are asked to practice these daily, initially in a half hour 'special time', but eventually during naturalistic opportunities throughout the day. At the end of the session, the therapist summarises progress achieved and successful strategies used and these are recorded in the written programme.

PACT-G was delivered by specifically trained speech and language therapists (SLTs), supervised by senior co-applicant SLTs. Therapists had prior experience of a wide range of children with ASD of differing developmental levels, understanding of developmental theories of autism and experience of implementing autism specific intervention strategies. In addition, therapists had skills in observation and working with parents and educators of children with ASD. Therapists attended an initial 2-day live training course delivered by the PACT therapy research team with post-course supervised practice with two clinical cases over three months to gain 80% fidelity on the PACT-G fidelity before commencing PACT-G trial cases. Fidelity was monitored throughout the trial with and feedback to local leads supervisors. Following recruitment and school consent, the therapist clinical lead at each site contacts the participants' school via the Head Teacher to discuss the intervention with relevant staff.

Introduction to the PACT-G Stages

The PACT-G intervention has 6 stages that follow a developmental hierarchy.

Stage 1 **Establishing Shared attention**

Stage 1 helps the parent/LSA to establish and maintain extended periods of mutual shared attention with the child. All dyads complete at least one session at Stage 1. At the start of the intervention some dyads will already be showing extended periods of mutual shared attention; in this case the focus of the single Stage 1 session is to ensure the parent/LSA has an understanding of the concept of shared attention and to highlight strategies the parent/LSA is already naturally using to promote it. For other dyads shared attention may only be fleeting and further sessions at Stage 1 are needed. This would include dyads where, in the initial assessment, the child is found to be at the very earliest stages of social development i.e. they do not demonstrate sufficient purposeful activity to permit mutual shared attention for shared focus and where a parent/LSA may need to scaffold or

demonstrate play and/or object use. Additional sessions at Stage 1 would also be indicated if the majority of mutual shared attention is initiated by the parent/LSA rather than the child; in this case the aim is to explore whether the amount of child-initiated shared attention can be increased. The parent/LSA learns sensitively to observe the child's focus, non-verbal and verbal signals and opportunities for shared attention.

Stage 2 **Synchronicity and sensitivity**
Here the emphasis is on parent/LSA sensitivity training, with a focus on the child's perspective and experiences. Parents/LSAs are encouraged to identify and use responses aimed at facilitating the child's social responsiveness, thereby (i) reducing asynchronous communication (mistimed responses which place a demand on the child) and (ii) increasing parent/LSA synchronous communication (responses adapted to the child's pace/ timing, commenting on and complementing the child's topic of interest). Directive responses and demands are replaced with synchronous responses, such as commenting on and acknowledging child intentions.

Stage 3 **Focusing on language input**
The parent/LSA selects and models language that accurately matches the child's communication competencies. Parent/LSA language and non-verbal gestures are carefully monitored and modified to be contingent with child comprehension and to increase the efficiency of child information processing. The parent/LSA is explicitly trained to respond to the child's non-verbal communication and to model complementary verbal responses that express the child's inferred communication intent.

Stage 4 **Establishing routines and anticipation**
This is a consolidation phase that aims to develop child verbal understanding, anticipation and participation using repetitive rhymes, predictable routine phrases and familiar interactive play.

Stage 5 **Increasing communication functions**
Communication acts are elicited by the sensitive use of communication "teasers" to provide opportunities for child initiation. For example, the parent/LSA may make use of pauses and gaps within familiar, predictable play situations which the child fills with social and verbal responses. Communicative teasers entice the child to initiate intentional communication. These are gradually extended to pose deliberate barriers and/or to introduce "sabotage" in situations where the parent/LSA makes obvious mistakes (e.g. offering an empty cup or unopened snack, or a puzzle/game with pieces missing). A range of pragmatic communication acts can be elicited in this way, including requesting, negating, directing and commenting.

Stage 6 **Expanding language and conversations**
The final phase involves elaboration. In addition to eliciting communication acts the parent/LSA provides simple expansions of the child's own play, actions and language, thus elaborating on the child's non-verbal, social and language repertoire.

Further details on measures

Diagnostic inclusion

Mullen Scales of Early Learning[1] **or** ***British Ability Scales;***[2] depending on child age and ability level. These are standard measures of early development which enable a developmental level of non-verbal abilities to be ascertained for inclusion criteria and allow characterisation of the cohort in relation to other autism treatment trials.

Social Communication Questionnaire (SCQ) Lifetime Version.[3] A brief (40-item) parent report screening measure that identifies characteristics associated with ASD. Items cover 3 subdomains: Reciprocal Social Interaction, Communication, and Restricted, Repetitive and Stereotyped Patterns of Behaviour. The 'lifetime' version of the SCQ refers to the entire developmental history of the child. The threshold score for the Core Autism categorisation used in this study is 22.

Secondary outcomes

Brief Observation of Social Communication Change (BOSCC) with researcher.[4-6] A researcher-coding of autism symptoms from videotaped child-adult interaction. It addresses the same autism symptom constructs as ADOS-2 but is designed to detect clinically meaningful symptom change in treatment studies. Codings combine symptom frequency, severity and atypicality on a 16-item, 0-5 scale (overall range 0-80). BOSCC is designed to be a standard treatment outcome measure for the autism field and is currently used in large funded trials in US and EU. The BOSCC has high to excellent inter-rater and test-retest reliability and has convergent validity with measures of communication and language skills. It demonstrates increased sensitivity to change over time compared to the ADOS-2 CSS in an observational study.[6] Three moderate-size RCTs applying the standard naturalistic BOSCC as an outcome measure reported small and not significant ES. [7-9] Applying the standard BOSCC coding scheme to a non-standard, structured parent-child interaction, one study found a large significant intervention effect.[10] For Module 1 BOSCC (non – or minimally verbal children) the recommended protocol consists of 4 minutes free play followed by 2 minutes of bubble play, and then repeated with a new set of toys. From this, 4 minutes of free play and 1 minute of bubbles was coded for each of the two segments. For Module 2 BOSCC (phrase speech) there was no recommended administration procedure developed at the start of PACT-G; thus, a play and conversation administration was agreed with the developers in which the children had 4 minutes of play followed by 2 minutes of conversation, which was then repeated with a new set of toys, before 2 minutes of bubbles.

Brief Observation of Social Communication Change (BOSCC) with parent and LSA.[4-6] Coded from video of child-parent play-session in home (baseline, 7 month midpoint, 12 month endpoint) and child-LSA in education setting (baseline, 7 month interim, 12 month endpoint); a measure of the intervention effect in the naturalistic settings in which intervention took place. BOSCC ratings here were made from the same video-capture as for the DCMA (see below); and to allow this, its administration deviated slightly from the recommended BOSCC protocol. For Module 1 BOSSC, each child had 8 minutes of free play with the adult with the same box of toys, followed by 2 minutes of bubbles, from this, 4 minutes of play and 1 minute of bubbles was coded for each BOSCC segment. For Module 2 BOSCC, parent and teacher BOSCCs had 8 minutes of play with the same set of toys, 4 minutes of conversation, and then 2 minutes of bubbles, based on a prototype 'verbal' BOSCC administration using Module 1 toys. It became apparent during the trial that the conversation element was too challenging for the majority of module 2 children and it was decided, in discussion with the BOSCC developers, to code the module 2 items from 4 minutes of free play and 1 minute of bubbles in each segment. The parent is asked to play naturally with the child, in free exploration of the toys and making it fun. The researcher prompts *"We would like you to play and talk together naturally as you would do normally at home. I will start the video when you and your child are settled and relaxed".* The LSA is asked to play naturally with the child, in free exploration of the toys and making it fun. *The researcher should ask the LSA about the typical style and type of interaction between adults and the child in daily routines, if there is an opportunity for natural interaction and make detailed notes. The researcher should instruct the LSA, if the child initiates an interaction with him/her,*

he/she should respond naturally and then encourage the child to continue playing with the toys available. The researcher prompts *"We would like you to spend time with the child, not directly teaching something but interacting, playing and talking together naturally as you would do in relaxed non-direct-teaching time and making it fun. I will start the video when you and your child are settled and relaxed".* For children with phrased / fluent speech only, after the play and bubble activities, a 4 minute adult-child conversation is recorded, where the adult is prompted "*Now you can talk together about what the child enjoys, his/ her favourite interests, activities he/she enjoys or things the child finds easy to talk about* ". This is recorded for 2 minutes. The adult is then prompted "Now you may like to include this picture in your conversation." This section is also recorded for 2 minutes.

Dyadic Communication Measure for Autism (DCMA) with parent and with LSA.[11] Coded from the same videos as for the BOSCC. This measure includes independent codes of adult communication (synchronous response) and child communication (child initiations). The DCMA synchronous response variable is defined as *the proportion of the adult's total communication acts that are synchronous with the child*, where a synchronous communication act is a comment or acknowledgement that follows in on the child's focus of play, actions, thoughts or intentions. Requests, directions, commands, questions and negations are not considered synchronous, even if related to the child's focus of attention. The DCMA child initiations variable is defined as *the proportion of the child's total communication acts that are initiations*, where initiations are verbal or non-verbal communication acts that serve to start an episode of interaction. This measure had proved sensitive in the original PACT mediation analysis and is used in PACT-G to test treatment effect and mediation in home and education settings.

Vineland Adaptive Behavior Scales. Parent and teacher versions (P/T-VABS).[12] The VABS includes domains of communication, daily living skills and socialisation; it has been used in numerous autism studies and measures child functional ability in the home and education settings.

MacArthur-Bates Communicative Development Inventories (Word and Gestures; Sentences and Grammar),[13] ***Receptive and Expressive One-word Picture Vocabulary Test,***[14] Pre-school Language Scale-5.[15] The overall language level measured by these standardised assessments supplements that of the measures of autism-specific communication included in the BOSCC and ADOS-2.

The Early Sociocognitive Battery of Very Early Processing Skills (ESB).[16,17] Assesses children's socio-cognitive skills (social responsiveness, joint attention, and symbolic comprehension).

Repetitive Behaviours Questionnaire (RBQ).[18] A 26 item parent questionnaire for assessing repetitive behaviours in children with ASD.

The Developmental Behaviour Checklist - Parent (2nd Edition; DBC-P)[19]***Disruptive / Anti-social and Anxiety Subscales***. A 96 item instrument used for the assessment of behavioural and emotional problems in young people aged 4-18 years with developmental and intellectual disabilities. It includes two subscales: the Disruptive / Anti-social and the Anxiety Subscale, (36 items) and is completed by a parent or carer.

Strengths and Difficulties Questionnaire (SDQ) – Parent and Teacher versions. [20] A 25-item brief measure of psychological wellbeing in 2-17 year olds completed by parents and teachers.

Child Health Utility 9D.[21] A paediatric measure of health related quality of life. It consists of nine items, rated on five levels (ranging from no problems to severe problems). The CHU9D is designed to be completed by children aged 7-17. Proxy completion by parents on behalf of their child, is also possible for younger/ developmentally disabled children.

Warwick & Edinburgh Mental-Wellbeing Scale.[22] Self- rated parental well-being questionnaire recommended by UK Department of Health as the preferred measure of mental wellbeing important to incorporate in studies of this kind.

Parental Self-efficacy (TOPSE).[23] A 48-item, self-report measure of parenting competence. It is a measure of possible change in parents' confidence in their ability to make a difference to their child's development. Completed at baseline and endpoint assessments.

Child and Adolescent Service Use Schedule (CA-SUS), incorporating School Service Use Schedule.[24] Developed to record therapies and service use accessed throughout participation in the study. Forms were adapted to young populations with autism in our PACT and PACT 7-11 studies.[25]

Working Alliance Inventory – Short Revised (WAI-SR).[26] A measure of engagement with therapy for parents and LSAs in intervention group only. For parents and LSAs, there is a simple rewording of the client and therapist versions of the WAI-SR, which has been validated and is now frequently used. Completed at 2 and 5 months into the intervention. To be analysed separately.

Composite Outcomes: Construction and Analyses

Language composite (MCDI, One-Word and PLS). Age effects regressed out of MB and One-Word raw scores. GSEM model then fitted with a baseline factor with six indicators (expressive and receptive scores from each of the three measures, those for the PLS scores allowing for censoring at the floor of the test using a tobit link) and similar for trial endpoint. Loadings were common for the same measure across baseline and endpoint factors. Endpoint factor was predicted by the baseline factor, site, gender, age-group and treatment allocation.

Emotional symptom composite (SDQ and Anxiety DBC). GSEM model fitted with baseline factor indicated by teacher and parent rated SDQ emotional scale and endpoint factor indicated by teacher and parent SDQ and DBC anxiety subscale, with common loadings over factors for the same measure. Endpoint factor was predicted by the baseline factor, site, gender, age-group and treatment allocation.

Repetitive Behaviour composite (Baseline SCQ Repetitive items - verbal rituals, stereotypic movements, and unusual preoccupations, Insistence on Sameness and Sensory Movements from RBQ). Within each of baseline and endpoint time-points, the two sub-scales of the RBQ were standardised and summed. The endpoint sum was regressed on the baseline sum, the sum of the 3 baseline SCQ items, site, gender, age-group and treatment allocation.

Adaptive behaviour composite (Parent and Teacher Vineland Socialisation and SDQ prosocial scales). GSEM model fitted with baseline factor indicated by teacher and parent Vineland and teacher and parent SDQ scales. Endpoint factor similarly defined, with common loadings over factors for the same measure. Endpoint factor was predicted by the baseline factor, site, gender, age-group and treatment allocation.

Parent Wellbeing composite (Warwick-Edinburgh and TOPSE scales). Simple sum of standardised total scores at baseline and endpoint, with analysis of covariance of outcome sum predicted by baseline sum, site, gender, age-group and treatment allocation.

Disruptive behaviour composite (SDQ externalizing and DBC disruptive subscales). Factors indicated by these two measures with common factor loadings at baseline and endpoint. Endpoint factor was predicted by the baseline factor, site, gender, age-group and treatment allocation.

Demographic, clinical and family language information
Relevant demographic and clinical information and details of home language(s) spoken with the child.

Statistical Analysis Plan and Primary outcome analysis

The ADOS-2 is a structured researcher-led assessment that maximises ascertainment of both social communicative competency and autism-related atypicality. Both tasks and scoring vary with the child's verbal ability according to the ADOS-2 module completed, and the same module was used at baseline as endpoint. Stratified by module, and for module 1 by child's level of language (to allow for the three items that differ for children with/without words), a module and language-level stratified regression of the endpoint ADOS-2 SA+RRB raw total score included treatment assignment, randomisation stratifiers (dummy variables for site, age-group and gender), baseline ADOS-2 score and any other baseline variables found to predict missingness. Residual plots were used to determine whether prior transformation of the ADOS-2 scores was required. Using the within group endpoint standard deviation, an effect size was calculated for each module stratum. A single pooled-across-modules estimate was calculated using a weighted mean, where the weights were the inverse of the variance of each stratum specific estimate. A confidence interval for this pooled estimate was obtained using 1000 boot-strap samples. To be consistent with the treatment main effects analysis, the test of difference in treatment effect by age group was based on the bootstrap p-value over 5000 replicates of the pooled within ADOS-2 stratum estimate of the treatment difference.

Endpoint ADOS-2 algorithm score was treated as a continuous variable, with the baseline score as a covariate and randomisation factors included. Three regression relationships were estimated, one for each of the strata defined by the ADOS-2 module (Mod-1 nonverbal, Mod-1 verbal, Mod 2) at endpoint. Regression residual plots were checked within each of the strata and showed no evidence of non-normality. A single estimate of the treatment effect was obtained by pooling the three stratum-specific Effect-Size estimates using the minimum-variance estimator. Univariate logistic regression showed that no baseline variables predicted missingness in the primary outcome data beyond those already be included in the prespecified model (stratifiers and baseline measures of the scale), so no others were included. Secondary analysis (see phase 3) is to report an optimal moderation index[27] including bias-correction from over-fitting to a finite sample.

Evolution of Primary Outcome selection

The first version of the SAP v0.0 was based on protocol V4 and was circulated to the DMC on 16/05/2017 after agreement from the TSC. This included the plan at that stage for a combined ADOS/BOSCC primary outcome. In subsequent discussion, the NIHR EME Funding Board required a change to revert to ADOS-2 as a unitary primary outcome. The SAP was subsequently updated accordingly, in a change also recorded in the trial protocol V6 and was circulated to the DMC for comment on 05/06/2019 as SAP V1. The SAP was then finalised after incorporating all comments from the DMC, TSC and the team as V1.2 which was signed off on 04/11/2019 before cleaning and eventual data lock on 31/01/2020.

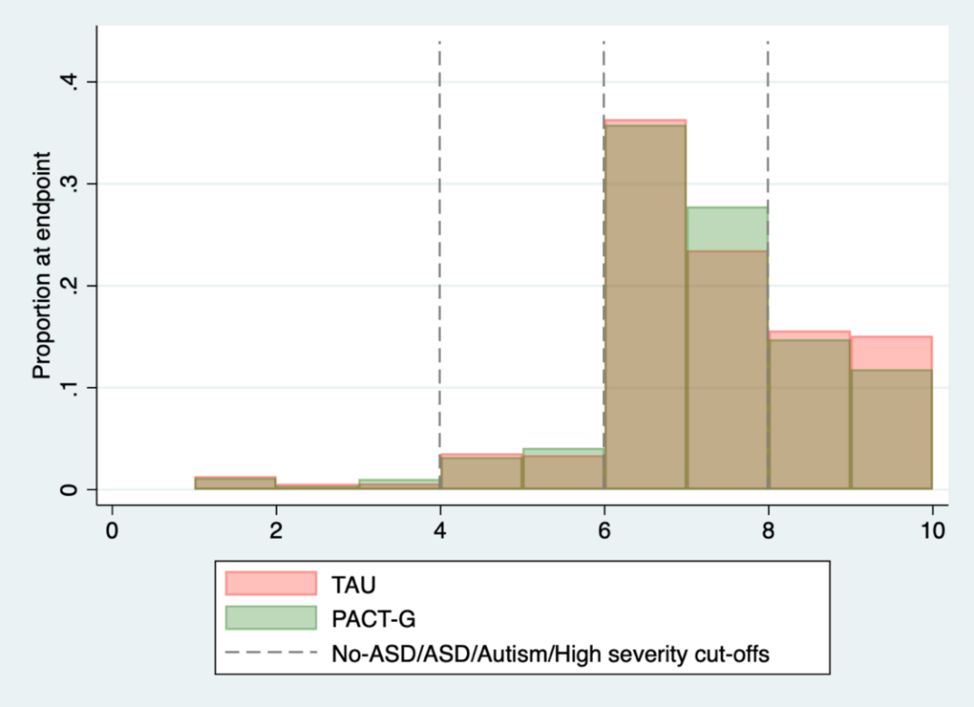

Figure S1 **Distributions of Calibrated Severity Score (CSS) Outcome**
Plotted by Trial-arm at endpoint for all participants as implied by the estimated effect on the ADOS-2 Algorithm Total score (mapping of scores done using Stata multiple imputation command using nearest neighbour matching of predicted scores and published 18-stratum total score to CSS algorithm).

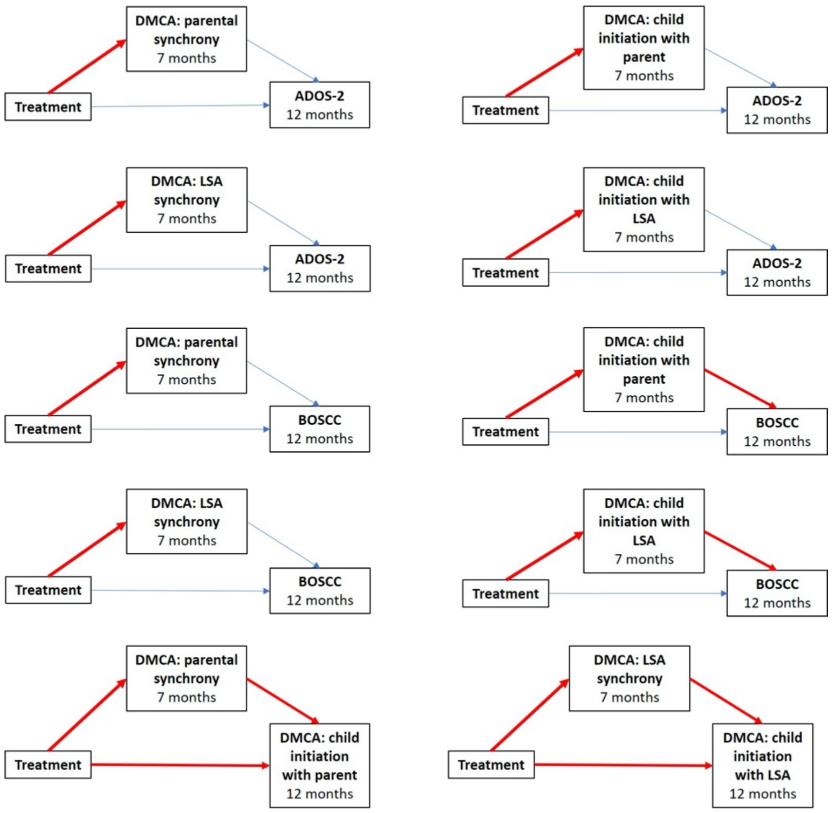

Figure S2 **Summary of the mediation analysis findings**
These need to be understood in relation to the hypothesised chain of effect (found in our previous studies) from intervention to: i) caregiver adult response to the child in the dyadic context, measured at 7 months; ii) child response to the caregiver adult in that same context, also at 7 months; iii) child response to another adult (researcher) in a generalised research context measured at 12 months.

Table S1 Participant withdrawals by arm

	TAU N (%)	PACT-G N (%)	Overall N (%)
**Did the participant withdraw from the study**			
No	121 (95.3)	119 (98.3)	240 (96.8)
Yes	5 (3.9)	2 (1.7)	7 (2.8)
Yes, and requested all data to be removed	1 (0.8)	0 (0.0)	1 (0.4)
**Reason for withdrawal**			
Participant no longer wishes to continue	5 (100.0)	0 (0.0)	5 (71.4)
Participant moved	0 (0.0)	1 (50.0)	1 (14.3)
Participant unhappy with progression of intervention	0 (0.0)	1 (50.0)	1 (14.3)

Table S2 Baseline comparison by Age Cohort

**Baseline**	**Pre-school N=151**	**School aged N=97**
ADOS-2 Module 1 non-verbal N=110	63	47
ESB soc resp median [range] (IQR)	0[0,12] (0 -1)	1[0,11] (0-3)
ESB jnt attn median [range] (IQR)	2[0,13] (0-5.5)	2 [0,17] (1-6)
Mullen visual raw score median [range]	23[16,34] (20-25)	26[16,41] (23-29)
>2 SD below standard norm t-score	97%	100%
Mullen fine motor raw score median	22[15,29] (19-24)	23[15,44] (20-25)
>2 SD below standard norm t-score	98%	100%
ADOS-2 Module 1 verbal N=77	52	25
ESB soc resp median [range] (IQR)	1.5[0,12] (0-3)	2[0,10] (0-3)
ESB jnt attn median [range] (IQR)	6[0,18] (3-9)	8[0,15] (6-11)
Mullen visual raw score median [range]	26.5[17,44] (24-	31[22,48] (28-34)
>2 SD below standard norm t-score	88%	96%
Mullen fine motor raw score median	27[19,44] (25-28)	29[19,46] (28-35.5)
>2 SD below standard norm t-score	92%	96%
ADOS-2 Module 2 N=61	36	25
ESB soc resp median [range] (IQR)	6[0,12] (2-8)	3[0,12] (1-8)
ESB jnt attn median [range] (IQR)	12 [0, 18] (7-15)	10[0,18] (8-12)
Mullen visual raw score median [range]	33[21,50] (30.5-42)	33[25,49] (30.5-43)
>2 SD below standard norm t-score	56%	92%
Mullen fine motor raw score median	30[23,44] (28-36)	33.5[21,47] (29.5-
> 2 SD below standard norm t-score	67%	96%
Over all ADOS-2 modules	151	97
ESB soc resp median [range] (IQR)	1[0, 12] (0-3)	2[0, 12] (0-4.5)
ESB jnt attn median [range] (IQR)	6[0, 18] (1-10)	6[0, 18] (2-10)
Mullen visual raw score median [range]	26[16, 50] (23-31)	29[16, 49] (25-32)
>2 SD below standard norm t-score	84%	97%
Mullen fine motor raw score median	26[15, 44] (22-28)	27[15, 47] (23-32)
>2 SD below standard norm t-score	89%	98%

Key: ADOS-2 = Autism Diagnostic Observation Schedule-2; Child Health Utility = Child Health Utility 9D; DBC = Dispruptive Behaviour Questionnaire; MacArthur CDI = MacArthur-Bates Communicative Development Inventories PLS = Preschool Language Scale; RBQ = Repetitive Behaviours Questionnaire; SCQ = Social Communication Questionnaire; SDQ = Strength and Difficulties Questionnaire; ESB=Early Socio-Cognitive Battery; WEMWBS = Warwick-Edinburgh Mental Wellbeing Scale

Table S3 Stage of therapy reached in PACT-G implementation in home and education

Endpoint Stage	Parents N (%)	LSA N (%)
1	12 (10)	24 (20)
2	29 (24)	27 (22)
3	44 (36)	42 (34)
4	17 (14)	15 (12)
5	15 (12)	9 (7)
6	4 (3)	4 (3)
**Total**	**121**	**121**

Table S4 **Nominated adult role per education setting type**

	PACT-G						TAU						
	Mainstream mursery	Specialist nursery	Mainstream primary school	Provision in mainstream	Special school (Mixed)	Special school (Autism only)	Mainstream mursery	Specialist nursery	Mainstream primary school	Provision in mainstream	Special school	Special school (Autism only)	Childminder
**Learning Support Assistant (N)**	27	3	24	1	20	17	27	1	18	2	31	13	0
Average level of qualification*	3.2	4.0	3.5	6.0	4.2	5.2	4.3	5.0	3.7	4.5	4.0	3.6	-
Average number of years in current role	6.7	9.7	7.0	0.0	10.2	4.5	6.5	3.0	8.0	2.0	8.7	5.2	-
Average number of years autism experience	4.8	17.3	3.9	0.0	12.8	4.3	5.5	3.0	3.9	3.0	9.1	7.0	-
**Teacher (N)**	3	0	0	0	3	1	5	0	4	0	1	0	0
Average level of qualification*	6.3	-	-	-	6.0	7.0	5.4	-	6.7	-	6.0	-	-
Average number of years in current role	2.0	-	-	-	2.3	2.0	10.6	-	3.3	-	8.0	-	-
Average number of years autism experience	1.3	-	-	-	11.7	5.0	7.2	-	2.3	-	8.0	-	-
**SENCo (N)**	3	0	0	0	0	0	9	0	0	0	0	0	0
Average level of qualification*	5.3	-	-	-	-	-	3.3	-	-	-	-	-	-
Average number of years in current role	6.3	-	-	-	-	-	7.8	-	-	-	-	-	-
Average number of years autism experience	11.0	-	-	-	-	-	5.9	-	-	-	-	-	-
**Other** (N)**	12	0	1	3	0	3	8	1	3	2	0	1	1
Average level of qualification*	3.3	-	4.0	5.0	-	4.3	3.8	3.0	4.4	5.5	-	5.0	3.0
Average number of years in current role	9.7	-	20.0	12.3	-	9.8	5.5	12.0	10.6	1.0	-	6.0	3.5
Average number of years autism experience	7.6	-	20.0	20.7	-	13.8	5.5	4.0	9.4	1.0	-	20.0	10.0

*Qualification level discriptions and equivalents can be accessed here: https://www.gov.uk/what-different-qualification-levels-mean/list-of-qualification-levels
**Other roles have been combined for the purposes of this table

Table S5 Number of families receiving non-PACT-G treatments during the 12-month trial period based on parental report

	PACT-G	TAU
**Home-based therapy (at least 4 sessions)**		
Applied Behaviour Analysis (ABA)	10	10
Number of sessions	Mean = 65 (5-236)	Mean = 89 (15-226)
Duration of sessions (Minutes)	Mean = 135 (120-300)	Mean = 153 (45-240)
CAMHS Behaviour Management	1	0
Number of sessions	6	0
Duration of sessions (Minutes)	45	0
Listening Therapy*	1	0
Number of sessions	500	0
Duration of sessions (Minutes)	15	0
Intensive Interaction	1	0
Number of sessions	12	0
Duration of sessions (Minutes)	240	0
**Speech and language provision in home or clinic settings**		
Picture Exchange Communication System	1	5
Number of sessions	3	Mean = 6 (1-12)
Duration of sessions (Minutes)	45	Mean = 47 (30-60)
Hanen	0	1
Number of sessions	0	8
Duration of sessions (Minutes)	0	150
EIBI / Verbal Behaviour	1	1
Number of sessions	12	60
Duration of sessions (Minutes)	360	3
Colourful Semantics	1	0
Number of sessions	2	0
Duration of sessions (Minutes)	40	0
Unnamed or eclectic therapy package	30	28
Number of sessions	Mean = 10 (1-50)	Mean = 12 (2-40)
Duration of sessions (Minutes)	Mean = 50 (15-90)	Mean = 58 (30-120)
Video-feedback involved**	1	6
**Autism-related parent training courses**		
Generic autism awareness/post-diagnostic course	30	34
Number of sessions	Mean = 6 (1-12)	Mean = 6 (1-20)
Duration of sessions (Minutes)	Mean = 144 (60-180)	Mean = 134 (60-240)
Communication/interaction	6	11
Number of sessions	Mean = 2.5 (1-8)	Mean = 7 (1-12)
Duration of sessions (Minutes)	Mean = 170 (60-420)	Mean = 153 (90-300)

Alternative and augmentative systems e.g., Makaton)	2	5
Number of sessions	Mean = 1	Mean = 3 (1-8)
Duration of sessions (Minutes)	Mean = 105 (90-120)	Mean = 126 (60-180)
Behaviour/sensory processing/emotion regulation	5	2
Number of sessions	Mean = 2.8 (1-8)	Mean = 7 (1-12)
Duration of sessions (Minutes)	Mean = 174 (120-240)	180
Non-specific/other parent training	8	5
Number of sessions	Mean = 4.75 (1-10)	Mean = 3 (1-7)
Duration of sessions (Minutes)	Mean = 197 (120-480)	Mean = 135 (120-180)

*This therapy consisted of the child listening to electronically modified sounds through headphones at various points of the day at home (e.g., pre- and post- school).
**This information was collected due to the similarities with- and relevance to- the video-feedback elements of the PACT-G intervention. Parents typically reported attending group-based programmes which were generic in nature, and that video-interactions were a 'one-off' or used as pre- post- record of progress. There was one family from London (in PACT-G trial arm) who reported that they had received PACT therapy.
*** Through an error in allocation-communication, one family in the TAU arm received the PACT-G therapy. Data from this family is reported and analysed as TAU.

Table S6 Number of children receiving non-PACT-G treatments in education settings reported by class-teacher

	PACT-G	TAU
SCERTS	1	0
Colourful Semantics	3	4
Music Therapy	1	1
Lego Therapy	2	1
Intensive Interaction	3	4
ABA / EIBI / Verbal Behaviour	0	2
Picture Exchange Communication System	12	4
Visual Supports / TEACCH	1	3
Makaton / Sign language	2	0
Unnamed or eclectic therapy package	36	50
Video-feedback involved*	4	4

*This information was collected due to the similarities with- and relevance to- the video-feedback elements of the PACT-G intervention.

Table S7 ADOS-2 treatment effects by module

ADOS-2	Unstandardised effect	Standard error	Standardised effect (95% CI)
Module 1 non-verbal N=96	-0.45	0.59	-0.13 (-0.48, 0.21)
Module 1 verbal N=82	-1.18	0.81	-0.27 (-0.63, 0.09)
Module 2 N=59	1.48	0.83	0.36 (-0.04, 0.75)

Table S8 Mediation of parent/LSA synchrony at 7 months on endpoint child initiations at 12 months

Outcome	Mediator	A path (SE); (95% CI), P-value	B path (SE); (95% CI), P-value	Ind. Effect (SE); (95% CI), P-value	Dir. effect (SE); (95% CI), P-value	Total effect (SE); (95% CI), P-value
Child initiations with parent at 12 months	Parent synchrony at 7 months	0.12 (0.02); (0.08, 0.16), <0.001	0.46 (0.08); (0.30, 0.62), <0.001	0.05 (0.01); (0.03, 0.08), <0.001	0.02 (0.02); (-0.02, 0.07), 0.341	0.08 (0.02); (0.03, 0.12), 0.001
Child initiations with LSA at 12 months	LSA synchrony at 7 months	0.11 (0.02); (0.07, 0.15), <0.001	0.31 (0.09); (0.14, 0.49), <0.001	0.03 (0.01); (0.01, 0.06), 0.003	0.02 (0.03); (-0.04, 0.07), 0.508	0.05 (0.03); (0.00, 0.11), 0.048

Table S9 **Mediation of treatment effects on researcher BOSCC at 12 months**

**Mediator**		**A path (SE); (95% CI), P-value**	**B path (SE); (95% CI), P-value**	**Ind. Effect (SE); (95% CI), P-value**	**Dir. effect (SE); (95% CI), P-value**	**Total effect (SE); (95% CI), P-value**
Parent Synchrony at 7 months	Module 1 N=191	0.12 (0.02); (0.07, 0.16), <0.001	0.09 (3.56); (-6.89, 7.07), 0.980	0.01 (0.42); (-0.81, 0.84), 0.980	-0.21 (1.12); (-2.39, 1.98), 0.854	-0.19 (1.02); (-2.19, 1.81), 0.854
	Module 2 N=57	0.13 (0.03); (0.07, 0.18), <0.001	-10.23(16.27); (-42.12, 21.66), 0.529	-1.30 (2.08); (-5.38, 2.77), 0.531	-0.02 (3.86); (-7.60, 7.55), 0.995	-1.28 (3.33); (-7.81, 5.25), 0.700
	OVERALL	0.12 (0.02); (0.09, 0.16), <0.001	-0.38 (3.48); (-7.20, 6.43), 0.912	-0.21 (0.50); (-1.18, 0.76), 0.671	-0.19 (1.07); (-2.29, 1.91), 0.858	-0.28 (0.97); (-2.19, 1.63), 0.773
Child Initiations with Parent at 7 months	Module 1 N=191	0.07 (0.03); (0.02, 0.12), 0.003	-6.82 (3.16); (-13.01, -0.63), 0.031	-0.51 (0.29); (-1.08, 0.07), 0.084	0.27 (1.03); (-1.75, 2.28), 0.796	-0.20 (1.02); (-2.20, 1.80), 0.843
	Module 2 N=57	0.11 (0.05); (0.02, 0.20), 0.023	-18.32 (9.61); (-37.16, 0.51), 0.057	-1.98 (1.32); (4.57, 0.61), 0.133	1.19 (3.48); (-5.63, 8.02), 0.732	-1.01 (3.42); (-7.73, 5.70), 0.767
	OVERALL	0.08 (0.02); (0.04, 0.13), <0.001	-7.94 (3.00); (-13.82, -2.06), 0.008	-0.78 (0.34); (-1.44, -0.11), 0.022	0.34 (0.99); (-1.59, 2.28), 0.730	-0.27 (0.98); (-2.18, 1.65), 0.784
LSA Synchrony at 7 months	Module 1 N=191	0.11 (0.02); (0.06, 0.16), <0.001	3.99 (3.22); (-2.33, 10.30), 0.216	0.43 (0.37); (-0.28, 1.15), 0.235	-0.62 (1.08); (-2.73, 1.49), 0.563	-0.18 (1.02); (-2.17, 1.82), 0.863
	Module 2 N=57	0.17 (0.04); (0.10, 0.24), <0.001	-41.82 (13.45); (-68.18,-15.45), 0.002	-6.92 (2.68); (-12.17,-1.67), 0.010	7.58 (4.44); (-1.13, 16.28), 0.088	-0.47 (3.86); (-8.03, 7.09), 0.903
	OVERALL	0.13 (0.02); (0.09, 0.17), <0.001	1.50 (3.13); (-4.64, 7.64), 0.633	-0.45 (0.46); (-1.34, 0.44), 0.324	-0.17 (1.05); (-2.22, 1.88), 0.873	-0.19 (0.98); (-2.12, 1.73), 0.843
Child Initiations with LSA at 7 months	Module 1 N=191	0.12 (0.03); (0.06, 0.19), <0.001	-4.23 (2.38); (-8.90, 0.44), 0.076	-0.52 (0.32); (-1.16, 0.12), 0.109	0.29 (1.05); (-1.76, 2.34), 0.784	-0.18 (1.02); (-2.18, 1.82), 0.857
	Module 2 N=57	0.14 (0.05); (0.04, 0.24), 0.008	-9.30 (9.23); (-27.38, 8.78), 0.313	-1.30 (1.39); (-4.02, 1.42), 0.350	-0.14 (3.66); (-7.32, 7.05), 0.971	-1.37 (3.41); (-8.05, 5.31), 0.687
	OVERALL	0.13 (0.03); (0.07, 0.18), <0.001	-4.55 (2.31); (-9.06, -0.03), 0.049	-0.67 (0.37); (-1.40, 0.06), 0.073	0.26 (1.00); (-1.71, 2.22), 0.800	-0.28 (0.98); (-2.20, 1.64), 0.774

Table S10 Therapist-rated criteria for intervention session adequacy

Exclusion deviations (U1 – U6)		Acceptable deviations (A1 – A12)	
U1	No targets set	A1	Additional person in during session
U2	Key adult not available	A2	Technical difficulties experienced
U3	It has not been possible for adult and therapist to watch a video of ACI	A3	Video is not watched simultaneously by therapist and adult but both have watched video
U4	Child doesn't take part in PCI	A4	Sibling present during session
U5	Sibling interfered in PCI to the extent that it disrupted the session	A5	Feedback was disrupted by child
U6	No therapy feedback	A6	PCI interrupted
777	Not available	A7	Additional full F2F sessions instead of remote (i.e. greater dosage)
888	Not done	A8	Skype/phone carried out in person
999	Unknown	A9	Review carried out after PCI
		A10	Missing data due to technical difficulty (however session fully delivered)
		A11	PACT approach required modification (e.g. teaching)
		A12	PCI video short
		A13	Other
		777	Not available
		888	Not done
		999	Unknown
**Exclusion deviations HSC**		**Acceptable deviations HSC**	
HSCU 1	Session under 30 minutes	HSCA 1	Additional person present
777	Not available	777	Not available
888	Not done	888	Not done
999	Unknown	999	Unknown

Table S11 Primary Outcome at Baseline and Endpoint by Age Group

ADOS-2	Baseline *mean (SD)*		Endpoint *mean (SD)*	
	**Treatment as usual**	**PACT-G**	**Treatment as usual**	**PACT-G**
Pre-school (n=97)	18.9 (4.2)	19.0 (3.3)	18.6 (4.2)	18.8 (3.9)
School aged (n=151)	18.4 (3.9)	18.7 (3.7)	16.8 (5.4)	16.8 (4.7)

Table S12 Primary Outcome at Baseline and Endpoint by Site

ADOS-2	Baseline *mean (SD)*		Endpoint *mean (SD)*	
	**Treatment as usual**	**PACT-G**	**Treatment as usual**	**PACT-G**
Site 1 (n=82)	19.0 (3.5)	19.1 (3.2)	16.7 (5.6)	17.0 (4.1)
Site 2 (n=82)	19.1 (4.5)	19.0 (4.0)	18.0 (3.9)	17.4 (3.3)
Site 3 (n=84)	17.6 (3.8)	18.2 (3.5)	17.8 (5.5)	18.3 (5.7)

References

1. Mullen EM. Mullen scales of early learning. Minnesota: American Guidance;1995.
2. Elliot CD, Smith P. British Abilities Scale-3 (BAS-3). *Windsor, Berks:* NFER-Nelson*;*2011
3. Rutter M, Bailey A, Lord C. Social Communication Questionnaire. Los Angeles: Western Psychological Services; 2003.
4. Carr T, Colombi C, MacDonald M, Lord, C. Measuring respond to intervention with the Autism Diagnostic Observation Schedule-Change (ADOS-C). Poster presented at Society for Research in Child Development Biennial Conference*,* Montreal, Canada; 2011.
5. Colombi C, Carr T, MacDonald M, Lord, C. Developing a measure of treatment outcomes: The Autism Diagnostic Observation Schedule-Change. Poster Presented at Society for Research in Child Development Biennial Conference, Montreal, Canada; 2011
6. Grzadzinski R, Carr T, Colombi C, McGuire, K, Dufek S, Pickles A, et al., Measuring Changes in Social Communication Behaviors: Preliminary Development of the Brief Observation of Social Communication Change (BOSCC). J Autism Dev Disord. 2016;46(7):2464–2479. Available from: https://doi.org/10.1007/s10803-016-2782-9
7. Divan G, Vajaratkar V, Cardozo P, Huzurbazar S, et al. The feasibility and effectiveness of PASS plus, a lay health worker delivered comprehensive intervention for autism spectrum disorders: pilot RCT in a rural low and middle income country setting. Autism Res. 2019;12(2):328-39. Available from: DOI: 10.1002/aur.1978
8. Fletcher-Watson S, Petrou A, Scott-Barrett J, Dicks P, Graham C, O'Hare A et al., A trial of an iPad™ intervention targeting social communication skills in children with autism. Autism. 2016;20(7):771-82. Available from: https://doi.org/10.1177/1362361315605624
9. Nordahl-Hansen A, Fletcher-Watson S, McConachie H, Kaale A. Relations between specific and global outcome measures in a social-communication intervention for children with autism spectrum disorder. Research in Autism Spectrum Disorders. 2016;29:19-29. Available from: https://doi.org/10.1016/j.rasd.2016.05.005
10. Gengoux G W, Abrams D A, Schuck R, Millan M E, Libove R, Ardel C M, et al., A pivotal response treatment package for children with autism spectrum disorder: An RCT. Pediatrics. 2019;144(3): p.e20190178. Available from: https://doi.org/10.1542/peds.2019-0178
11. Pickles A, Le Couteur A, Leadbitter K, Salomone E, Cole-Fletcher R, Tobin H, et al. Parent-mediated social communication therapy for young children with autism (PACT): long-term follow-up of a randomised controlled trial. Lancet. 2016;388:2501-2509.
12. Sparrow SS, Cicchetti DV, Balla DA. Vineland adaptive behavior scales. 2nd ed.*Oxford:* NCS Pearson, Inc;2005.
13. Fenson L, Marchman V, Thal D, Dale P, Reznick S, Bates E. MacArthur Communicative Development Inventories: User's guide and technical manual. 2nd ed. Baltimore, MD:Brookes;2007.
14. Brownell R, Receptive and expressive one-word picture vocabulary tests (ROWPVT-4, EOWPVT-4). 4th ed. San Antonio:Pearson Education;2010.
15. Zimmerman, IL, Steiner, VG, Pond E. Preschool Language Scales (PLS-5). 5th ed. San Antonio, TX:Pearson;2011.
16. Chiat S, Roy P. Early predictors of language and social communication impairments at 9-11 years: A follow-up study of early-referred children. J Speech Hear Res. 2013;56:1824-36. Available from: https://doi.org/10.1044/1092-4388(2013/12-0249)
17. Roy, P., & Chiat, S. (2019). The Early Sociocognitive Battery: A clinical tool for early identification of children at risk for social communication difficulties and ASD? International Journal of Language and Communication, 54, 794–805.
18. Honey E, McConachie H, Turner M, Rodgers J. Validation of the repetitive behaviour questionnaire for use with children with autism spectrum disorder. Res Autism Spectr Disord. 2012;6:355–64. Available from: https://doi.org/10.1016/j.rasd.2011.06.009
19. Einfeld, SL, Tonge BJ. Manual for the Developmental Behaviour Checklist: Primary Carer Version (DBC-P) & Teacher Version (DBC-T). 2nd ed. Clayton, Melbourne: Monash University Centre for Developmental Psychiatry and Psychology;2002.
20. Goodman R. The strengths and difficulties questionnaire: a research note. J Child Psychol Psychiatry.1997;38(5):581–86. Available from: https://doi.org/10.1111/j.1469-7610.1997.tb01545.x

21. Stevens K. Valuation of the child health utility 9D index. PharmacoEconomics. 2012;30:729–47. Available from: https://doi.org/10.2165/11599120-000000000-00000
22. Tennant R, Hiller L, Fishwick R, Platt S, Joseph S, Weich S et al., The Warwick-Edinburgh Mental Well-being Scale (WEMWBS): development and UK validation. Health and Quality of Life Outcomes. 2007;5(63). Available from: https://doi.org/10.1186/1477-7525-5-63
23. Kendall S, Bloomfield L. Developing and validating a tool to measure parenting self-efficacy. J Adv Nurs. 2005;51(2):174–81. Available from: https://doi.org/10.1111/j.1365-2648.2005.03479.x
24. Barrett B, Byford S, Sharac J, Hudry K, Leadbitter K, Temple K et al., Service and wider societal costs of very young children with autism in the UK. J Autism Dev Disord. 2012;42(5):797-804. Available from: DOI: 10.1007/s10803-011-1306-x
25. Green J, Aldred C, Charman T, Le Couteur A, Emsley R A, Grahame V, et al. Paediatric Autism Communication Therapy-Generalised (PACT-G) against treatment as usual for reducing symptom severity in young children with autism spectrum disorder: study protocol for a randomised controlled trial. Trials. 2018;19(1). Available from: https://doi.org/10.1186/s13063-018-2881-3
26. Hatcher RL, Gillaspy JA. Development and validation of a revised short version of the Working Alliance Inventory. Psychother Res. 2006;16(1):12–25. Available from: https://doi.org/10.1080/10503300500352500
27. Kraemer HC. Discovering, comparing, and combining moderators of treatment on outcome after randomized clinical trials: a parametric approach. Stat Med. 2013;32(11):1964-73. Available from: https://doi.org/10.1002/sim.5734

THE PAEDIATRIC AUTISM COMMUNICATION TRIAL - GENERALISED (PACT-G)
TRIAL PROTOCOL

1. GENERAL INFORMATION

	ISRCTN: 25378536<br>NIHR REFERENCE: 13/119/18
**Trial Office:**	The PACT-G Office<br>Room 3.312<br>Jean McFarlane Building<br>The University of Manchester<br>Oxford Road<br>Manchester M13 9PL
**Email:**	kathy.leadbitter@manchester.ac.uk<br>PACT-G@manchester.ac.uk
**Website:**	http://www.pact-g.org/
**Funders:**	NIHR/MRC EME Programme<br>University of Southampton<br>Alpha House<br>Enterprise Road<br>SO16 7NS<br>Telephone: 02380594303<br><br>Department of Health<br>Room 132 Richmond House<br>79 Whitehall<br>London, SW1A 2NS<br>Telephone: 02072103824<br>(Excess treatment costs subvention)
**Sponsor and Monitor:**	Manchester University NHS Foundation Trust,<br>Dr. Lynne Webster,<br>Central Research Office,<br>Nowgen Building,<br>Grafton Street<br>M13 9WL<br>Telephone: 01612764125<br><br>**Collaborating Institutions**<br>Central Manchester University Hospitals NHS Foundation Trust<br>University of Manchester<br>Newcastle University<br>Guy's & St Thomas' NHS Foundation Trust (Evelina Children's Hospital)<br>Institute of Psychiatry, Psychology and Neuroscience, King’s College, London
**Project team**	*Trial Office*<br>Professor Jonathan Green - Chief Investigator<br>Dr Kathy Leadbitter – Project Manager<br>Helen Morley – Trial Manager<br>Claire Bennett - Trial Administrator
**Principal Investigators**	Dr Catherine Aldred

Consultant Speech & Language Therapist
Children's and Young People's Disability Partnership
Kingsgate House
Wellington Road North, Stockport
SK4 1LW
0161 204 4135

Professor Tony Charman
Chair in Clinical Child Psychology
Institute of Psychiatry, Psychology & Neuroscience
Department of Psychology
Box PO77,
Henry Wellcome Building
De Crespigny Park,
Denmark Hill,
London
SE5 8AF
Telephone: 0207 848

Professor Ann Le Couteur
Professor of Child & Adolescent Psychiatry
Institute of Health & Society,
Level 3, Sir James Spence Institute,
Royal Victoria Infirmary,
Queen Victoria Road,
Newcastle upon Tyne
NE1 4LP
Telephone: 0191 282 1398

Prof Richard A Emsley
Professor of Medical Statistics and Trials Methodology
Department of Biostatistics and Health Informatics
Institute of Psychiatry, Psychology and Neuroscience, Room S 2.03
De Crespigny Park
London
SE5 8AF
Telephone: 0207 848 0724

Professor Patricia Howlin
Emeritus Professor of Clinical Child Psychology
Institute of Psychiatry, Psychology & Neuroscience Department of Psychology
Box PO77
Henry Wellcome Building,
de Crespigny Park,
London
SE5 8AF

Professor Neil Humphrey
Research Director/Professor: Psychology of Education
University of Manchester
Ellen Wilkinson Building, Oxford Road
M13 9PL
Telephone: 0161 275 3404

Dr Kathy Leadbitter
Research Associate
MA, MSc, PhD
University of Manchester

Rm 3.316 Jean McFarlane Building
Oxford Road
Manchester
M13 9PL
Telephone: 0161 3067964

Professor Helen McConachie
Professor of Child Clinical Psychology
Newcastle University,
Institute of Health and Society
Level 3 Sir James Spence Institute,
Royal Victoria Hospital,
Queen Victoria Road,
Newcastle upon Tyne
NE1 4 LP
Telephone: 0191 282 1396

Dr Jeremy R Parr
Clinical Senior Lecturer and Consultant, Paediatric Neurodisability
Institute of Neuroscience,
Framlington Place,
Newcastle University
NE1 9DU
Telephone: 0191 282 5966

Professor Andrew Pickles
Prof. of Biostatistics and Psychological Methods; Director of King's Clinical Trials Unit
Institute of Psychiatry, Psychology and Neuroscience, Room S 2.10
De Crespigny Park
London
SE5 8AF
Telephone: 0207 848 0724

Dr Vicky Slonims
Senior Consultant Speech & Language Therapist
Guy's & St Thomas' NHS Foundation Trust (Evelina Children's Hospital)
Newcomen Centre at St Thomas'
2nd Floor, Becket House, 1 Lambeth Palace Road,
London SE1 7EU
Telephone: 0207 188 6238

Dr Vicki Grahame
Consultant Clinical Psychologist
Northumberland, Tyne and Wear NHS Foundation Trust
Walkergate Park,
Benfield Rd.,
Newcastle, NE6 4QD

Collaborators

Trial Statistician

Professor Andrew Pickles
Director of King's Clinical Trials Unit
Institute of Psychiatry, Psychology and Neuroscience, Room S 2.10
De Crespigny Park
London
SE5 8AF

Telephone: 0207 848 0724

Trial Steering Committee

Professor Stuart Logan (Chair)
Professor Anne O'Hare
Professor Liz Pellicano
Ms Louisa Harrison (parent representative)
Ms Kellie Bell (parent representative)
Professor Jonathan Green (CI)

Data Monitoring and Ethics Committee

Professor Paul Ramchandani
Professor Amanda Farrin
Professor Jacqueline Barnes
Professor Andrew Pickles (PI Statistician)

Clinical Research Network (CRN)
Trial adopted by the NIHR CRN in March 2016

PACT-G will be conducted in accordance with the principles of GCP and applicable UK regulatory requirements

2. PROTOCOL AMENDMENTS

The following changes have been made to the protocol.

Old Version	*New version*	*Date*	*Amendment*
**1.0**	**2.0**	**06.10.2016**	**Addition of parent questionnaires in sections 8.2 and 8.3**
**2.0**	**3.0**	**18.11.2016**	• **Finalise list of assessments in section 8.2 and add schedule table in 8.3 Add safeguarding to exclusion criteria**<br>• **Update score on SCQ inclusion**<br>• **Update data collection and CTU information**<br>• **Add exclusion criterion for non-agreement by schools**
**3.0**	**4.0**	**02.02.2017**	• **Throughout protocol: Change to timing of midpoint & endpoint assessments**<br>• **Section 6.2: Addition of exclusion criterion**<br>• **Section 7.2: clarity about a maximum of 12 therapist-LSA sessions**<br>• **Section 8.2: deletion of Epworth Measure of Daytime Sleepiness**<br>• **Section 8.2: Inclusion of Developmental Behaviour Checklist**<br>• **Section 15: Information added about ethical approvals**
**4.0**	**5.0**	**01.09.2017**	• **Section 6.2: Further details added about p-level inclusion criterion**<br>• **Section 7.2: Updated details of treatment protocol**<br>• **Section 8.1: Updated Primary Outcome Measure**<br>• **Updated Consort Diagram**<br>• **Section 9: Statistical Analyses -Changes as a result of proposed change to primary outcome**<br>• **Section 15 – updated Ethical Approvals**
**5.0**	**6.0**		• **Update to contact details**<br>• **Section 8.1: Updated Primary Outcome Measure**<br>• **Section 9: Statistical Analyses -Changes as a result of proposed change to primary outcome**<br>•

3. TRIAL SUMMARY

Background: The evidence base for early intervention in autism shows that behaviours proximal to the intervention delivered (e.g. dyadic interaction measures) are amenable to change.However, it has been difficult to generalise treatment gains successfully acquired from one context into another, and no studies to date have demonstrated improvement in general autism symptom severity (difficulty with the generalisation of acquired skills is a key problem in autism).

Aim: This proposal tests an intervention designed systematically to promote generalisation of previously demonstrated clinic-assessed treatment gains into home and school contexts. It includes a detailed mediation analysis building on our previous work and a mechanism study that will enable for the first time a detailed approach to understanding the generalisation of therapy-acquired skills across contexts.

Hypotheses: 1) The intervention will show the added efficacy and cost-effectiveness of preschool and school-age autism outcomes in home, school and research settings compared to treatment as usual; 2) There will be an increase in the generalisation of acquired communication across contexts and persons, shown by mediation and the mechanism study.
Design: Three site two parallel group randomised controlled trial of the experimental treatment plus treatment as usual (TAU) versus TAU alone. Initial pilot first stage with pre-specified progression criteria.
Population: Children 2-11 years meeting criteria for core autism on gold-standard measures. Interventions: The experimental intervention builds on our clinic-based MRC Preschool Autism Communication Treatment model (PACT), delivered with the primary caregiver using methods that gave maximal intervention effect on child social communication, combined with additional targeted theory and evidence based strategies designed to enhance the generalisation of this effect into naturalistic home and education contexts (details below). The control intervention will be treatment as usual.
Primary outcome: Autism symptom outcome, researcher assessed in standardised setting. Secondary outcomes: Context-related autism symptoms, child interaction with parent or teacher, language and reported functional outcomes in home and school settings. Outcomes measured at baseline and 12 month endpoint in all settings with interim interaction measurements (7 months) to test mediation.
Sample: 244 (122 intervention/122 TAU; 82/site).
Primary Analysis will test for between-group change in primary outcome using analysis of covariance plus planned subgroup analysis by age-group stratifier and test of moderation. Mechanism analysis will use regression models to test for mediation of parent-child interaction on primary outcome.
Duration: 42 months, with 6 month start-up, 6 months pilot stage with progression criteria, 24 months for the main trial, and 6 months analysis and write up. 244 cases will be recruited over months 9-26 (4.8/month/site). This is very feasible given that our previous PACT trial with the same inclusion criteria recruited 2.6/month, and recruitment here will be from three times the population pool due to expanded age criteria (<15% of available cases during this period from population estimates).

4. BACKGROUND INFORMATION

The Paediatric Autism Communication Trial – Generalised (PACT-G) builds on the work of Pre-school Autism Communication Trial (PACT) conducted in Manchester, London and North East England between 2006 and 2009. [1]

4.1 Existing research

Intervention research in autism spectrum disorder (hereafter 'autism') has recently accelerated, with studies across a range of interventions considered in recent NICE guidance,[2] Cochrane[3] and other reviews.[4,5] The pattern of findings across a number of interventions is for reproducible moderate to good effects on targeted proximal outcomes such as improvement in interaction and communication in the treatment context[1,6] but weaker evidence for generalisation of treatment effect to broader symptom change and functional outcome.[4] The problem of generalising from 'proximal' intervention effects to wider symptom and functional change is currently a key current challenge for autism treatment research.[5,7]

4.2 Theoretical background

The capacity to generalise acquired skills flexibly across contexts is a central feature of successful developmental learning but a major problem for individuals with autism.[8] Typical development, for instance of language or social skills, depends on children being able to generalise skills acquired in one setting (and with one communicative partner) for use in another (and with other partners). There are a number of theories as to why children with autism should find this so difficult, ranging from their learning style,[9] lack of predictive information coding,[18] probabilistic thinking[19] or weak neural connectivity.[10] However, in practice, from the behavioural/psychological treatment literature there are well-established strategies for enhancing the generalisation of acquired skills.[8,11] *Parent mediated learning*, providing the same dyadic cues for the child across different contexts, is one plausible approach to helping overcome the generalisation difficulties in autism,[12] and *Naturalistic learning,* in which the learning takes place within the functional context in which the skills are actually needed, provides another important approach. Working with children in their natural environments is now highlighted as best practice for early intervention.[13] PACT-G systematically builds on this background and on analysis from our previous trial (described below), by incorporating parent- and teaching staff- mediated intervention strategies within the naturalistic learning contexts of home and education into the PACT intervention model. These additional, evidenced-based, strategies are designed to improve generalisation

of the proximal treatment effects demonstrated in our original study to wider symptom change and functional impact in other environments.

4.3 Evidence from the PACT trial

The PACT therapy is a parent-mediated social communication intervention tested in the most substantial trial yet undertaken in the autism field,[1] and has been subsequently subjected to detailed mediation analysis of treatment process.[14 and supplementary information] The intervention was mainly delivered in clinic, although parents were asked to undertake practice at home. Compared to treatment as usual (TAU), PACT showed a rapid and substantial impact on the targeted immediate outcome of parental communication style (enhanced parental communicative synchrony with the child) (ES 1.22 (95% CI 0.85,1.59) at 13 month endpoint and 1.44 at 6 months). This change in parental synchrony strongly mediated (>70%) a substantial improvement in the child's communication initiations with the parent (ES 0.41 (0.08, 0.74) at 13 months, 0.5 at 6 months). Treatment effect on autism symptoms (measured within researcher-child interaction) was attenuated (ES 0.24 (-0.59, 0.11)) at endpoint, but analysis showed that the endpoint symptom change that did occur was strongly mediated (73%) by the enhanced child communication initiation with parent at midpoint (Figure 1).[14]

Figure 1: Outcome and mediation in the PACT trial[1,14]

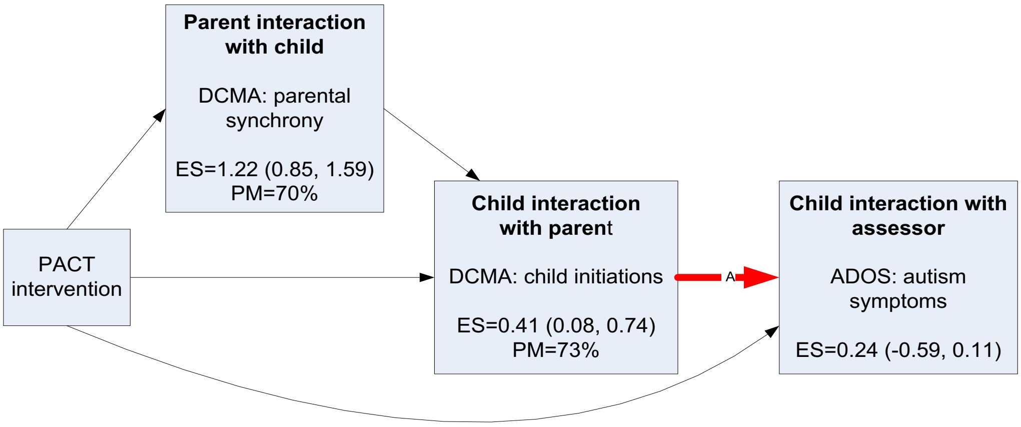

ES: effect size of PACT intervention on that variable; PM: proportion mediated of PACT intervention on next step in the causal pathway; DCMA, Dyadic Communication Measure in Autism; ADOS, Autism Diagnostic Observation Schedule (both measures described below). Red arrow shows where the PACT-G targets generalisation.

These results illustrate the attenuation of treatment effect on generalisation across context (parent-child to researcher-child interaction in different contexts), but also demonstrate a causal chain of mediation influence across these contexts. As such, they reflect both the difficulty of but also the possibility for generalisation. Thus, while the symptom outcome change at endpoint in PACT was modest, the change that did occur was strongly mediated by the significant change in child dyadic communication with parent. This suggests that the symptom change itself was meaningful in direction and would be increased if the transmission pathway from child initiation to symptoms could be enhanced.[14] The PACT trial was not able to further test the generalisation of the child's acquired competencies into the everyday environment of home or education using blinded measures. However, non-blinded parent-rating showed highly significant generalised treatment gains in both receptive language (OR 3.4 (1.48 to 7.79)), expressive language (OR 1.63 (0.76 to 3.51)) and social communication (OR 2.49 (1.27 to 4.89)),[1] further suggesting the potential for generalisation of treatment effect into naturalistic contexts.

4.4 Rationale for current study

As Indicated above that generalisation of child acquired dyadic communication skills in PACT was facilitated by having the same interaction partner across environments but weakened when the interaction partner and context changed. Building on this evidence, in this current trial we test a significantly modified PACT intervention including extensions of the intervention procedure into the naturalistic contexts of home and education setting, aimed at enhancing treatment effects in these generalised contexts and with a range of

partners. We will test the impact of this enhanced intervention in both the home and education settings; and the cumulative impact of this in enhancing overall symptom outcomes.

A further modification is to extend the application of the intervention into the primary school years. Autism intervention studies to date have been largely limited to episodic interventions, usually in pre-school. However, communication skills continue to emerge and develop beyond the pre-school years[15] and social communication skills in the early school age period are strong predictors for later development.[16] The persisting and significant impairments in social interaction and communication among children with autism argue strongly for a developmentally sustained approach to intervention into middle childhood in affected children.

The ***mechanism study*** will build on the understanding gained from the design and mediation analysis in the original PACT trial as above[14] by assessing the mediators and outcomes in the different generalisation contexts, and thus provide a unique and innovative opportunity to further understand the processes and facilitation of symptom change in autism.

5. RESEARCH OBJECTIVES:

Objective 1 - Testing the efficacy of the PACT-G intervention
To test whether the extended PACT social communication intervention protocol, using targeted enhancement strategies within home and education settings, improves transmission of treatment effect to:
a) Researcher-assessed autism symptom outcome.
b) Autism symptoms and functional adaptation in home and education settings.
This objective will be tested using blinded measures maximising ability to detect meaningful change (see measures below) and evaluated by analysis at trial endpoint.

Objective 2 - Mechanism analysis to illuminate generalised skill acquisition in autism.
The mechanism analysis will use the experimental trial to illuminate core processes of generalisation of specific acquired competencies in autism across context.
(i) We will build on the mediation analysis from our previous PACT Trial (see above) to test mediation of the generalised treatment effect in home and school.
(ii) We will test how effects in naturalistic contexts may combine to enhance transmission of effect to research-assessed symptoms in a standardised test setting.
We will use the pre-specified measures of mediation, which were successful in our previous MRC PACT trial.

6. RESEARCH DESIGN

Efficacy study: Three-site, two-group, randomised controlled trial of the experimental treatment plus treatment as usual (TAU) compared to TAU alone. Children between the ages of 2 – 11 years with defined autism will be recruited to the trial in the local areas following referral via clinical specialists, education professionals and consented databases. After consent families will be randomised on three sites around the UK to receive either the PACT-G social communication intervention in addition to the treatment as usual (PACT-G) or treatment as usual (TAU) alone**.** Assessments are administered on entry (baseline) to the trial, at the 7 month midpoint and at the 12 month endpoint.

There will be an initial 6-month external pilot stage, with pre-specified progression criteria to the full trial. The pilot will be a feasibility, acceptability and recruitment study on 24 cases (8 at each site) using the full baseline and eligibility assessment battery for all cases and the first phase of the intervention for 12 cases. There will be particular focus on the novel aspects of the intervention and research protocol, including the home-based generalization, education buy-in and implementation**.**

Mechanism study: An embedded mechanism study to test mediation hypotheses and illuminate the basic science of generalisation impairments in autism.

6.1 Randomisation procedure and methods to minimise bias

Research staff will confirm eligibility and obtain consent. Baseline assessment will be undertaken prior to treatment assignment.

Randomisation will be done through the web-based King’s College Clinical Trials Unit randomisation service. Allocation will be by stratified block randomisation with randomly varying block sizes controlling for treatment

centre, age strata (2-4 years, 5-11 years), severity and gender. Each case will be assigned a participant ID number and treatment allocation emailed separately to the treatment centre therapists.

There will be separate clinical and research leads at each site and separate training and supervision structures. Researchers will be housed separately from staff involved in delivery of the PACT-G intervention.

Mid- and endpoint research interviews and assessments will be conducted so as to avoid inadvertent divulging of information that could infer treatment status. The assessment suite and materials used will be quite different in type and location to that used for the therapy intervention avoiding any familiarity effect for children in the treatment arm. The primary outcome and mediation measures are coded from videotape, by researchers at the other sites, trained to high levels of reliability and blinded to intervention allocation. A random 10% of assessments will be double rated for reliability by an external blinded expert. All other researcher assessments are also blinded; parent and teacher questionnaires/interview measures non-blinded.

All therapy sessions are videotaped. Variability due to therapist effects will be minimised by frequent clinical supervision and checks on continuing therapist fidelity against the treatment manual; a minimum of 5% of randomly selected sessions for each therapist will be formally coded for fidelity over the course of the study by independent clinicians using the model successfully used in PACT.

6.2 Study population*:*

Inclusion criteria:

- Age 2 -11 years
- Clinical diagnosis of Autism Spectrum Disorder
- Meeting criteria for autism on the Autism Diagnostic Observation Schedule-2nd Edition (ADOS-2)[17] and Scoring ≥15 (school-aged) and ≥12 (preschool) on the Social Communication Questionnaire (SCQ).
- (For children who are aged 5 years) Between levels P3 and P8 for the English curriculum (as reported by relevant professionals0. P levels are designed to be used for pupils with learning disability. P3 communication skills would indicate that a child is beginning to use 'intentional communication'. P8 represents a level up to but not beyond a language age equivalent of 4 years in a typically developing child.)
- Parents with sufficient English to potentially participate in the intervention and who speak English to their child at least some of the time.

Exclusion criteria:

- Sibling with autism already in the trial
- Participation in PACT-G pilot phase
- Children ≤12 months non-verbal age equivalent level,
- Epilepsy not controlled by medication,
- Severe hearing or visual impairment in parent or child,
- Current severe learning disability in the parent, or current severe parental psychiatric disorder
- Current safeguarding concerns or other family situation that would affect child / family participation in the trial.
- No agreement to participate from child's education setting
- Children with an identified genetic disorder that would impact on ability to participate or affect validity of data; eligibility to be determined by PIs on a case-by-case basis)

7. TRIAL TREATMENTS:

7.1 Treatment Principles

PACT-G therapy is an enhancement of the original clinic-based PACT therapy. This is a 'parent-mediated' therapy in which caregivers are coached, using video-feedback, to interact with their child using evidence-based strategies that facilitate communication development in the child. Optimal interaction with a sensitive and responsive communication partner (such as the parent/caregiver) increases communication and social interaction skills in the child. In the original PACT trial this approach was found to be very effective in increasing the

quality of parental communicative responses to the child, which in turn led to increased child-initiated communications with the parent.

PACT-G therapy retains these effective elements but adds new features to aid the generalisation of the child's newly acquired skills into other settings, recognising that such generalisation is a particular problem in autism. PACT-G therapy encourages generalisation of skills by extending the therapy into the home and school settings, by integrating the parental techniques into daily routines and play and by widening the range of adults involved in training to include education staff in addition to parents / carers. The therapy begins with the parent at home then extends into the educational setting. Flexibility in timing is built in to fit with school terms, with an overlap to allow for essential supported joint collaboration with parent and education staff.

PACT-G therapy has also been modified to incorporate recent advances in research, focusing on specific strategies to enhance the child's response to adult-directed shared attention and to develop object interest and play. These are important precursors to the early stages of language development[18,19] and have been shown to moderate treatment response in recent social communication early autism trials.[20] Further modifications allow more individual differentiation so that intervention begins at a point appropriate to the child's initial level of object interest and social engagement.

PACT-G therapy, in common with the original PACT therapy, takes a staged approach, which is based on theoretically informed child developmental progression and strategies for establishing essential foundation skills, such as shared attention. Parents and education staff are helped to recognise and facilitate child motivated play and increase their synchrony and sensitive responding (stages 1-2) with verbal comments on child action and play. Middle stages (stages 3-4) of PACT-G develop language comprehension and expression through commenting on the child's activity, language 'mapping' and modelling, and encourage child communication initiations through the use of anticipation and other eliciting techniques. For children who make the most progress, later stages (stages 5-6) encourage language expansion and conversation. PACT-G therapy is appropriate for pre-school and also primary school age children who have severe autism. Some children are likely to be at the earliest stages of communication development making the early developmental PACT-G stages focusing on shared attention, adapted parent responding and eliciting child communication initiation appropriate. Other children may be verbally fluent making appropriate the later PACT-G stages, which focus on language understanding, expression, language expansions and conversation.

7.2 Treatment Protocol

The sequence of delivery of the PACT-G intervention is set out visually in figure 2.

***Parent sessions*:** Based on what was found to be most effective in the original PACT trial, parents will receive 12 intervention sessions. Prior to starting the intervention, a home visit is conducted to introduce the intervention to the parents, explore the family context and set expectations. Generally, the therapy sessions start in the home but can also be undertaken in the clinic. Subsequent sessions alternate between home based sessions and Skype/telephone-delivered consultation. Delivery is flexible in accordance with the needs of the family. This approach will assist generalisation of new skills development in the home setting. Clinical and research experience indicates that these session formats are popular with parents.[21] Each parent session begins with a discussion of progress made since the last session. The parent and therapist then watch a either a 10-minute video, made by the therapist of the parent and child in play or a 1-2 minute parent-made video of a home based practice session routine. The therapist facilitates the parent to identify actions that lead to child communication and to adopt PACT-G strategies in their interaction with the child. Parents are assisted to set goals for themselves, based on the interaction strategies discussed. The parent and therapist discuss the opportunities to practice these strategies each day outside of therapy sessions and parents are asked to make time to practice them for half an hour a day daily.

***Education setting sessions*:** In most cases, therapy in the educational setting begins after the parent has commenced therapy. The start times and duration of education-based therapy are flexible to fit around the school term schedule. In the education setting PACT-G sessions will be delivered to trained learning support assistants (LSA), who are staff with a specific remit to assist children with special educational needs to access the curriculum and broader school based activities. LSAs and other education staff receive an initial training session to introduce them to PACT-G therapy. The education-based intervention then consists of therapist-LSA sessions that mirror the therapist-parent sessions in the home. Videos are made of the LSA and the child and are used to coach the LSA in the use of appropriate PACT-G strategies. The LSA then implements these with the child daily in class time. There are a maximum of 12 therapist-LSA sessions over 6 months, alternating in-

school visits and skype/telephone consultations. PACT-G strategies are expected to be integrated in a complementary way with other communication strategies that may already be in use in the school.

***Collaboration between parent and educational staff*:** Importantly, the separate therapeutic work with parents and LSAs described above will be supplemented with a schedule of joint parent-LSA meetings to support the work and ensure consistent use of strategies across home and education settings. This will be key to successful generalisation. The meetings will use the manualised technique of ‘Home-School Conversation’ (HSC) [22,23]. Meetings are structured around ‘explore’, ‘focus’, ‘plan’ and ‘review’ stages, which allow the LSA and parent to share experiences and maximize intervention consistency. HSC is validated and shown to be highly effective in motivating parents and schools.[22,23]

Figure 2: Intervention and assessment timeline

Month	0<br>Baseline	1	2	3	4	5	6	7	8	9	10	11	12<br>Endpoint
Assessment	BOSCC<br>ADOS												BOSCC<br>ADOS
Assessment	BOSCC<br>DCMA							BOSCC<br>DCMA					BOSCC<br>DCMA
Intervention with parent			Initial home visit<br>12 intervention sessions (home based sessions and telephone/skype support sessions)<br>HSC sessions in school						HSC sessions continue for the period of the school intervention, until endpoint<br>The number will vary depending on term times but with a minimum of 4 sessions				
Assessment	BOSCC<br>DCMA							BOSCC<br>DCMA					BOSCC<br>DCMA
Intervention with education					Initial LSA in-school training visit*<br>Up to 12 intervention sessions (school alternating with Skype/telephone support)<br>incorporating HSC meetings with parents**						HSC sessions continue until final assessment		

*Start of education element accommodates school terms **Home-School Conversation – see text.

7.3 Training and Fidelity of Treatment

Training in the PACT-G therapy will be conducted centrally by the lead speech and languagetherapists, who will undertake overall co-ordination of the therapy in the trial and will organise quarterly across-site therapist meetings. Therapists will be regularly supervised by the lead speech and language therapists in each site. All therapy sessions will be videotaped and 5% of randomly selected tapes will be independently rated using the PACT Fidelity Rating Scale at regular intervals across the trial period.

Therapists in the trial will not be treating any TAU patients.

Therapists and research staff will be trained in practices that minimise noncompliance and drop-out. Therapy compliance and receipt of other interventions outside of the protocol will be monitored.

7.4 Treatment as Usual

The control intervention will be treatment as usual (TAU). We have detailed information on TAU in the pre-school population from the group's previous work on the MRC PACT trial and in older children from the PACT 7-11 early school study.[24] Data on services received will be collected.

8. ASSESSMENTS AND PROCEDURES

8.1 Primary outcome

Autism Diagnostic Observation Schedule (ADOS-2)[17 25] The standard autism diagnostic symptom measure with good external validity to long-term outcomes. Measured within researcher-child interaction using a standardised set of social presses, video-recorded for later coding. The scoring metrics of ADOS have been modified in line with the 2013 DSM-5, with social communication and repetitive behavior symptom domains combined into a unitary total symptom score (Social Affect + Restricted and Repetitive Behavior Overall Total raw score). Recent studies [26, 27] have demonstrated the ability of the ADOS to measure treatment effects; in the PACT trial sustained 6 years after treatment end.

8.2 Other measures

Brief Observation of Social Communication Change (BOSCC) with researcher.[28,29,30] BOSCC is a researcher-coding of autism symptoms from videotaped child-adult interaction. It addresses the same autism symptom construct as ADOS (which was used in the original PACT trial) but is designed to better detect clinically meaningful symptom change in treatment studies, with codes combining symptom frequency, severity and atypicality on a 16-item, 0-5 scale (overall range 0-80). BOSCC is designed to be a standard treatment outcome measure for the autism field and is currently used in large funded trials in US and EU. It shows high inter-rater agreement[28] and increased sensitivity to treatment change compared to ADOS (BOSCC d=0.64 compared to parallel ADOS d=0.42 in a recent 12 month observational intervention study).[31]

Mullen Scales of Early Learning[33] ***or British Ability Scales***;[34] depending on child age and ability level. These are standard measures of non-verbal early development which
enables a developmental level of non-verbal abilities to be ascertained for inclusion criteria and to allow characterisation of the cohort in relation to other autism treatment trials.

Social Communication Questionnaire (SCQ) Lifetime Version[35] Standard instruments, to be used for diagnostic inclusion.
The SCQ is a brief (40-item) parent report screening measure that identifies characteristics associated with ASD. Items cover 3 subdomains: Reciprocal Social Interaction, Communication, and Restricted, Repetitive and Stereotyped Patterns of Behaviour. The 'lifetime' version of the SCQ refers to the entire developmental history of the child.

Brief Observation of Social Communication Change (BOSCC) with parent and LSA[28,29,30] Coded from video of child-parent play-session in home (baseline, 7 month midpoint, 12 month endpoint) and child-learning support assistant in school (baseline, 7 month interim, 12 month endpoint); measure of intervention effect in naturalistic settings in which intervention took place.

Dyadic Communication Measure for Autism (DCMA) with parent and with LSA[1,36] Coded from video of the child-parent play-session at home (baseline, 7 months midpoint, 12 month endpoint) and child-learning support assistant play-session in school (baseline, 7 months interim, 12 month endpoint). This measure includes independent codes of parental communication (synchrony) and child communication (initiations). This measure proved sensitive in the original PACT mediation analysis and will be used in PACT-G to test treatment effect and mediation in home and education settings.

Vineland Adaptive Behavior Scales. Parent and teacher versions (P/T-VABS)[37] The VABS includes domains of communication, daily living skills and socialisation, and has been used in numerous autism studies. It will be a measure of functional gains by the child in the home and education settings.

MacArthur-Bates Communicative Development Inventories (Word and Gestures; Sentences and Grammar)[38]***; and Receptive and Expressive One-word Picture Vocabulary Test***[39]***; and Pre-school Language Scale-5***[40]***.*** The overall language level measured by these standardised assessments supplements that of the measures of autism-specific communication included in the BOSCC and ADOS.

Warwick & Edinburgh Mental-Wellbeing Scale[41] Parent rated well-being questionnaire recommended by DoH as the preferred measure of mental wellbeing important to incorporate in studies of this kind.

Child and Adolescent Service Use Schedule (CA-SUS)[24] Developed to record service use and adapted to young

populations with autism in our PACT and PACT7-11 studies.[1]

Working Alliance Inventory – Short Revised (WAI-SR)[42] Therapeutic Alliance questionnaire; measure of engagement with therapy for parents and learning support assistants in intervention group only; For parents and LSAs, there is a simple rewording of the client and therapist versions of the WAI-SR, which has been validated and is now frequently used. Completed at 2 and 5 months into the intervention.

Family History Interview (FHI)[43] Measure of the Broader Autism Phenotype (BAP) in parents. Completed at midpoint assessment.

Strengths and Difficulties Questionnaire (SDQ) – Parent and Teacher versions The SDQ Short Form (Goodman, 1997)[44] This is a 25-item brief measure of psychological wellbeing in 2-17 year olds. In PACT-G, it will be completed by both parents and teachers.

Tool to Measure Parental Self-efficacy[45] A 48-item, self-report measure of parenting competence. It is a measure of possible change in parent's confidence in their ability to make a difference to their child's development. Completed at baseline and endpoint assessments.

The Developmental Behaviour Checklist - Parent (2nd Edition; DBC-P)[46] ***Disruptive / Anti-social and Anxiety Subscales***. The DBC-P is a 96 item instrument used for the assessment of behavioural and emotional problems in young people aged 4-18 years with developmental and intellectual disabilities. It is completed by a parent or carer. In PACT-G we will use two subscales: the Disruptive / Anti-social and the Anxiety Subscale. This constitutes 36 items.

Child Health Utility 9D[47] A paediatric measure of health related quality of life. It consists of nine items, each responded to with one of five levels (ranging from no problems to severe problems). The CHU9D is designed to be completed by children aged 7-17. Proxy completion is also possible for younger/ developmentally disabled children. In PACT-G parents will be asked to complete this questionnaire on behalf of their child.

Repetitive Behaviours Questionnaire[48] – 26 point questionnaire; the RBQ is one of the most commonly used measures for assessing repetitive behaviours in children with ASD

Demographic, language and service use information – we will collect relevant demographic information and details of languages spoken with the child; as well as information about therapies and services accessed throughout participation in the study.

(For possible future ecollection – not currently acquired: ***Family History Interview (FHI)***[43] Measure of the Borader Autism Phenotype (BAP) in parents)

8.3 Schedule of assessments

The table below shows the full schedule of assessments

	Measure
Eligibility	
	ADOS-2
	Mullen Scales of Early Learning (pre-school children)
	British Ability Scales (school-age children)
	SCQ Lifetime version
Baseline	BOSCC – Researcher
	BOSCC/DCMA – Parent
	BOSCC/DCMA – LSA
	Vineland – Parent Interview
	Vineland - Teacher Survey
	Early Socio-Cognitive Battery - ESB

	Receptive and Expressive One-Word Picture Vocabulary Test
	Repetitive Behaviour Questionnaire
	Warwick & Edinburgh Mental Wellbeing Scale
	MacArthur-Bates Communicative Development Inventories (Word & Gestures; Sentences & Grammar)
	Strengths and Difficulties Questionnaire – Parent
	Strengths and Difficulties Questionnaire – Teacher
	Tool to Measure Parental Self-Efficacy
	Child Health Utility 9D
	Key Information and Demographics
	Clinical Information and Service Use
	School Service Use Form
	Family Language Interview
**7-month Home/Parent**	BOSCC/DCMA – Parent
	Trial Status Form
**7-month LSA/School**	BOSCC/DCMA – LSA
**Endpoint**	ADOS-2
	Repetitive Behaviour Questionnaire
	BOSCC – Researcher
	BOSCC/DCMA – Parent
	BOSCC/DCMA – LSA
	Preschool Language Scale-5
	Receptive and Expressive One-Word Picture Vocabulary Test
	Vineland - Teacher Survey
	Vineland – Parent Interview
	Warwick & Edinburgh Mental Wellbeing Scale
	MacArthur-Bates Communicative Development Inventories (Word and Gestures)
	Developmental Behaviour Checklist – Parent (Disruptive/Antisocial & Anxiety Subscales)
	Strengths and Difficulties Questionnaire – parent
	Strengths and Difficulties Questionnaire – teacher
	Tool to Measure Parental Self-Efficacy
	Changes to Key Information and Demographics
	School Service Use Form
	Child and Adolescent Service Use Schedule (CASUS)
	Child Health Utility 9D

PACT-G Consort Diagram

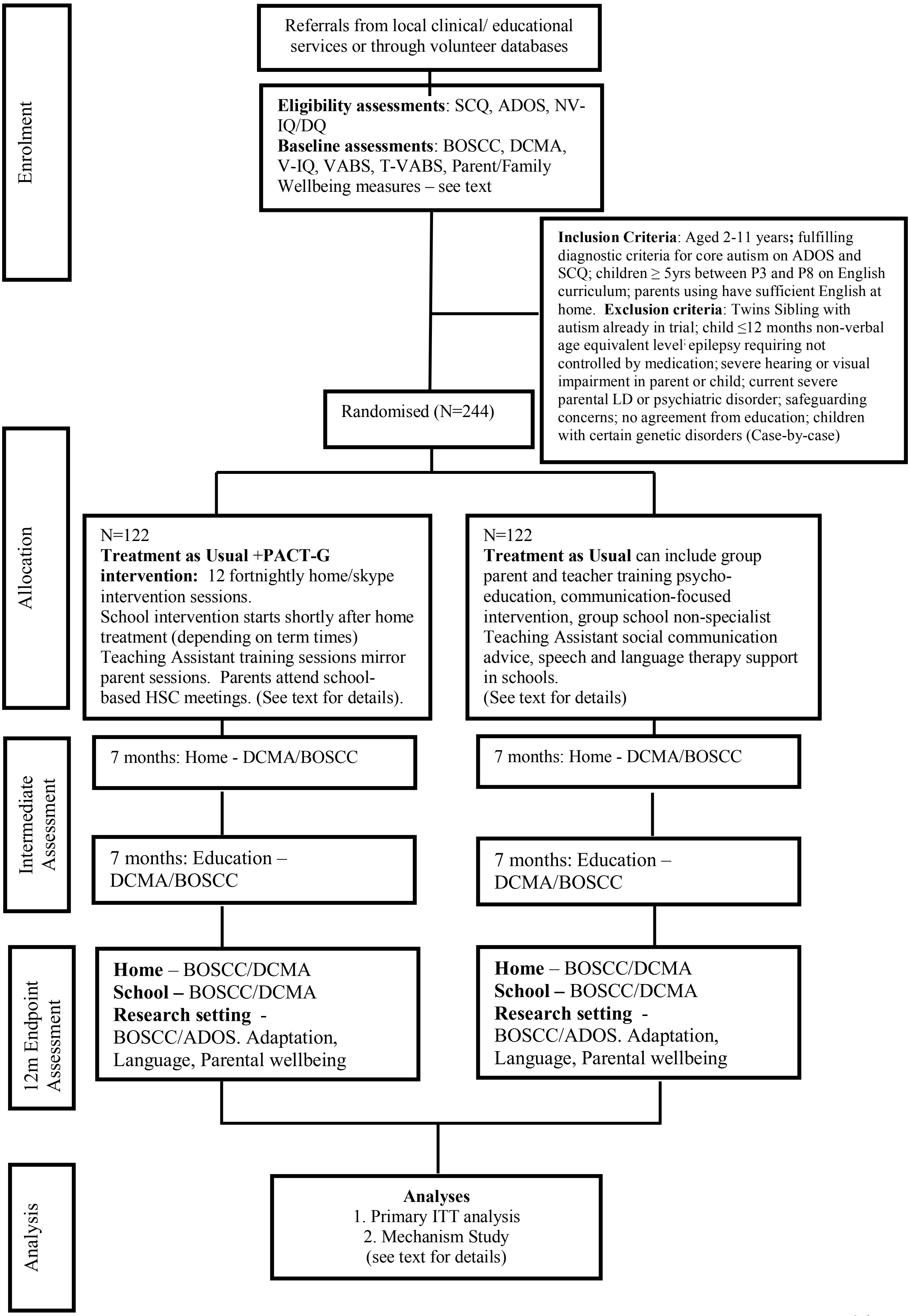

9. STATISTICAL ANALYSIS

9.1 Sample Size Calculations

Our PACT trial showed an effect of ES 1.22 (0.85, 1.59) on parental synchrony (DCMA), which mediated 70% of the ES 0.41 (0.08, 0.74) on Child communication, which in turn mediated 72% of the ES 0.24 (0.59, 0.11) on symptom outcome (ADOS). The intervention strategies in PACT-G are specifically targeted to enhance generalisation of the child communication to increase primary outcome effects in home, education and research settings. Therefore we expect the ES for the symptom outcome to be substantially above 0.24 and clinically meaningful (see above). Power was calculated using the sampsi command in Stata, for an analysis using ANCOVA with alpha=.05, with pre and post measures correlated .67 (from PACT trial). With 110 cases followed up in each group (70/70 preschool and 40/40 school-age) 80% power is retained for ES=0.28 and 90% power for ES=0.33. Allowing for 10% attrition (compared to 4% in PACT) we propose to recruit 244 families (82/site - 52 pre + 30 school-age).

9.2 Analysis Plan

All statistical analyses will be carried out using the latest version of Stata[49] or MPlus (see http://www.StatModel.com). In accordance with CONSORT statement for non-pharmacological interventions, we will report all participant flow. Descriptive statistics of recruitment, drop-out and completeness of interventions will be provided.

Phase 1 - Efficacy Analysis.
Analysis of all treatment effects will be undertaken after 12 month outcome measures are completed. The main efficacy analysis will be via intention-to-treat including all participants, with no planned interim analysis for efficacy or futility. Baseline characteristics will be presented by randomised group without formal statistical tests. We will test the primary hypothesis for between-group difference in the outcome ADOS Total score using regression, stratified by ADOS module, covarying by baseline ADOS total and dummy variables for site, gender and age group. Standard residual diagnostics will be applied and skew minimising transformations adopted where required. An overall effect size will be calculated pooling stratum specific estimates for strata defined by the ADOS module, weighted by their precision, using a 95% confidence interval estimated from 5000 bootstrap replicates.

The secondary outcomes will be analysed in a similar way but without stratification by ADOS module. A forest plot of effect sizes for primary and secondary outcomes will be presented. A test of homogeneity of effect-size for the ADOS and BOSCC will be reported.

The primary paper will report a test of homogeneity of effect for the primary outcome in pre-school and school-age children. A secondary paper (see phase 3) will report an optimal moderation index[50] including bias-correction from over-fitting to a finite sample.

Phase 2 - Mechanisms Evaluation. Mediation analysis[14] gave detailed insight into an attenuated generalization in the original PACT trial across change in person, task and context (as above and Figure 1). In PACT-G we enhance generalisation *into home* by keeping parental dyadic cues constant but increasing functionally relevant interaction contexts; and *into education* by enhancing relevant communication with education staff (LSA). The mechanism study will investigate the mediation process in this model and through that illuminate key basic knowledge about generalisation in autism. Some of the pathways of interest are illustrated in Figure 3. If the efficacy analysis shows significant between group differences in the mediators (DCMA at home (path a) and education setting (path c)), then we will use parametric regression models to:

1. test for mediation of the intervention on ADOS outcome with researcher through DCMA at home (paths a,e,f);
2. test for mediation of the intervention on ADOS outcome in education setting through DCMA at education (paths c,d,f);
3. test for mediation of intervention on DMCA in education setting through DCMA at home (paths a,b,c);
4. use structural equation modelling to examine multiple pathways through DCMA at home and education setting to generalisation on the primary outcome of ADOS (paths a-f)

We will also repeat these four steps using researcher BOSCC as the outcome variable in place of the ADOS. Since all the measures are continuous, the indirect effects are calculated by multiplying relevant pathways and bootstrapping is used to produce valid standard errors for the indirect effects. All analyses will adjust for baseline measures of the mediators (DCMA), outcome (ADOS/BOSCC) and putative measured confounders. Mediation analyses are potentially biased by measurement error in mediators and hidden confounding between mediators and outcomes; we will build on our previous methodological and applied work in this context to include repeated measurement of mediators and outcomes to account for classical measurement error[14] and baseline confounding. We will investigate the sensitivity of the estimates to these problems and that of unmeasured confounding using instrumental variable (IV) methods[51] with baseline covariate by randomization interactions as potential instruments.[51]

Figure 3: Key mediation pathways to be tested in PACT-G mechanism study.

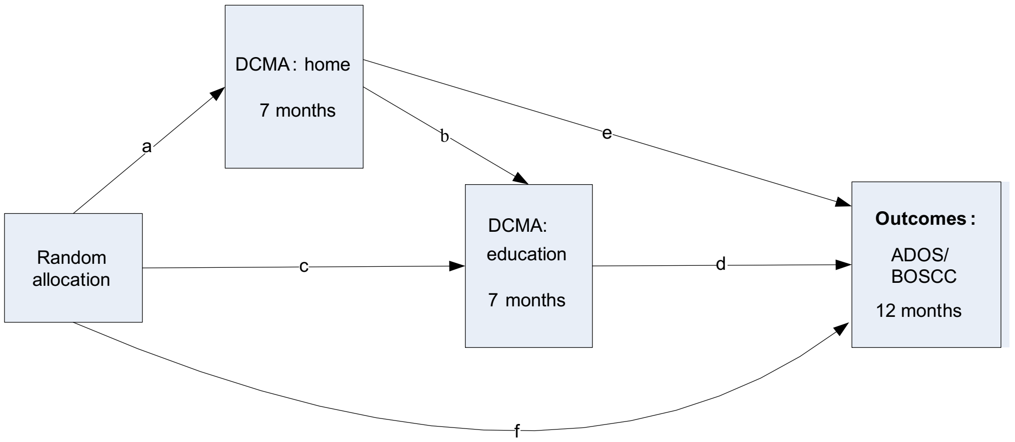

Treatment compliance in the education setting is likely to be more variable than the high levels achieved with parents. We will estimate a complier average causal effect (CACE) estimate using instrumental variable methods, considering the extent of education-setting opt-in as a measure of compliance and randomisation as the instrumental variable.

Phase 3 - Moderation and subgroups: We will test whether the mediation analysis is consistent across the two age-groups by testing for moderation of paths a-f by age-group stratifier (including interaction terms or performing a multiple group analysis in the structural equation model). We will test "*moderated mediation*" on our pathway from intervention to interaction with an unfamiliar assessor, extending our understanding of generalisation processes in autism. The heterogeneity of autism is well-recognised and as such offers numerous potential moderators of treatment effects (e.g. language level, restricted and repetitive behaviour, functional impairment). We will examine an extended list of moderators using bias correction/cross-validation methods (we are currently comparing the performance of alternative penalisation methods using the original PACT data and will apply our findings from this to the current cohort) to identify robust evidence for moderation and for a moderation index, both on the overall effect and also along the steps of the mediation pathway. Due to our proposed sample size, the power of analysis possible here will be unprecedented in autism research (and to our knowledge in other areas of psychological intervention in childhood).

10. TRIAL SUPERVISION

This study will be sponsored by Central Manchester University Hospitals NHS Foundation Trust and subject to normal governance arrangements.

10.1 Trial Steering Committee

We will form a trial steering committee (TSC), which will include an independent chair, parent representatives from the PACT 7-11 cohort and other service user representatives, as well as national organisations such as the National Autistic Society, which has strongly supported the PACT and PACT 7-11 studies from the outset. This steering committee will be consulted on the final design of the follow-up, techniques for ascertainment and the focus for measurement. The TSC shall meet once prior to the commencement of the trial and annually thereafter.

10.2 Data Monitoring and Ethics

There will be an independent data monitoring methods committee (DMEC).

10.3 Project management group

The project management group will be chaired by Professor Green and consist of the Principal Investigators and senior researchers on the trial, the Trial Manager and other invited members as necessary. It will meet at least quarterly, with additional tele or video-conferencing as necessary.

10.4 Adverse events

We will collect information about adverse events; as well as recording adverse events in the standard way, we will include events particularly relevant to this trial, such as significant changes in family or school situation.

11. DATA HANDLING AND RECORD KEEPING

All data in the trial will be anonymised. A central master file will be held by the trial manager at the Room 3.312, Jean McFarlane Building, The University of Manchester, Oxford Road, Manchester M13 9PL. This will contain the key linking anonymised trial name to personal details. This eCRF pack will be backed up securely within the web based data entry service of Kings College CTU. All data will be entered into the Kings web based secure MACRO database, which has a full audit trail and appropriate quality control will be carried out during the trial and before the database lock.

12. DATA ACCESS AND QUALITY ASSURANCE

Primary analysis of the data will take place in Kings College, London and the University of Manchester by the trial statisticians, Professor Andrew Pickles and Professor Richard Emsley, and Chief Investigator, Professor Jonathan Green. Other members of the team will also have access to data and will undertake analysis as appropriate and necessary. Any arrangements for other researchers in the general field to have access to the primary data will be negotiated separately and COREC informed.

The data will be stored in the Academic Department of Child Psychiatry, University of Manchester. Paper copies will be stored centrally in secured cabinets. Electronic data will be stored within the Kings College CTU secure data storage facility and on the central computer of the Department of Child & Adolescent Psychiatry, University of Manchester. The custodian will be Professor Jonathan Green, Chief Investigator of the study.

13. PUBLICATION

The results of the research will be targeted for publication in peer-reviewed journals of general and special interest. There will also be a general dissemination programme for families including participants co-ordinated through our collaborators in the National Autistic Society. Individual feedback for participants will be through the regular trial newsletter.

14. FINANCE

NIHR Research funding - £1,699,810.24
DH funding for excess treatment costs - £857,870.00

15. ETHICAL APPROVAL

The trial has received ethical approval from the North West – Greater Manchester Central Research Ethics Committee on 28.01.16 (ref: 15/NW/0912).

Notices of substantial amendment	Number 1	Approved 04.05.16
	Number 2	Approved 30.11.16
	Number 3	Approved 22.12.16
	Number 4	Approved 07.03.17

16. REFERENCES

1. Green J, Charman T, McConachie H, Aldred C, Slonims V, Howlin P, Le Couteur A, Leadbitter K, Hudry K, Byford S. Parent-mediated communication-focused treatment in children with autism (PACT): a randomised controlled trial. *The Lancet* 2010; 375(9732): 2152-60.
2. National Institute for Health and Care Excellence. Autism: the management and support of children and young people on the autism spectrum, Clinical guideline 170, 2013. National Institute for Health and Clinical Excellence site. Available at: http://guidance.nice.org.uk/CG170. Accessed Jan 31, 2013.
3. Oono IP, Honey EJ, McConachie H. Parent-mediated early intervention for young children with autism spectrum disorders (ASD). Cochrane Database Systematic Review 2013 Issue 4. Art. No. CD009774. DOI:10.1002/14651858.CD009774.pub2.
4. Charman T. Early identification and intervention in autism spectrum disorders: Some progress but not as much as we hoped. *International Journal of Speech & Language Pathology* 2014; 16(1): 15-18.
5. Lord C, Wagner A, Rogers S, et al. Challenges in evaluating psychosocial interventions for autistic spectrum disorders. *Journal Autism and Developmental Disorders* 2005; 35: 695–708.
6. Kasari C, Paparella T, Freeman S, Jahromi LB. Language outcome in autism: Randomized comparison of joint attention and play interventions. *Journal of Consulting and Clinical Psychology* 2008; 76(1): 125-37.
7. Warren Z, McPheeters ML, Sathe N, Foss-Feig JH, Glasser A, Veenstra-Vanderweele J. A systematic review of early intensive intervention for autism spectrum disorders. *Pediatricsm* 2011; 127:e1303-11.
8. Schreibman, L. Intensive behavioral/psychoeducational treatments for autism: research needs and future directions. *Journal of Autism and Developmental Disorders* 2000; 30: 373-378.
9. Qian N, & Lipkin, RMA. Learning-style theory for understanding autistic behaviors. *Frontiers in Human Neuroscience* 2011; 5: 77.
10. Just MA, Keller TA, Malave VL, Kana RK, & Varma S. Autism as a neural systems disorder: a theory of frontal-posterior underconnectivity. *Neuroscience and Biobehavioral Reviews* 2012; 36: 1292-1313.
11. Stokes TF, & Baer DM. An implicit technology of generalization. *Journal of Applied Behavioral Analysis* 1977; 10: 349–367.
12. Paul R. Interventions to improve communication in autism. *Child Adolescent Psychiatric Clinic of North America* 2008; 17(4):835-56, ix-x. doi:10.1016/j.chc.2008.06.011.
13. Carl J, Dunst CJ, Raab M, Trivette CM. Characteristics of Naturalistic Language Intervention Strategies. *Journal of Speech-Language Pathology and Applied Behaviour Analysis* 2012;(5): 3-4, 8-16
14. Pickles A, Harris V, Green J, Aldred C, Mcconachie H, Slonims V, Le Conteur A, Hudry K, Charman T. Treatment mechanism in the MRC preschool autism communication trial: Implications for study design and parent-focussed therapy for children. *Journal of Child Psychology and Psychiatry* 2014. doi:10.1111/jcpp.12291
15. Wodka EL, Mathy P, Kalb L. Predictors of phrase and fluent speech in children with autism and severe language delay. *Pediatrics* 2013; 131:e1128 –e1134.
16. Howlin P, Moss P, Savage S, Rutter M. Social outcomes in mid to later adulthood among individuals diagnosed with autism and average nonverbal IQ as children. *Journal of the American Academy of Child & Adolescent Psychiatry* 2013; 52(6): 572-81.
17. Lord C, Rutter M, DiLavore PC, Risi S, Gotham K, Bishop S. Autism Diagnostic Observation Schedule, Second Edition (ADOS-2) Manual (Part I): Modules 1–4. Torrance, CA: Western Psychological Services; 2012.
18. McDuffie AS, Lieberman RG, Yoder PJ. Object interest in autism spectrum disorder: a treatment comparison. *Autism* 2012; 16(4): 398-405.
19. Siller M, Sigman M. Modelling longitudinal change in the language abilities of children with autism: parent behaviors and child characteristics as predictors of change. *Developmental Psychology* 2008; 44(6):1691-704.
20. Carter AS, Messinger DS, Stone WL, Celimli S, Nahmias AS, Yoder PJ. A randomized controlled trial of Hanen's 'More Than Words' in toddlers with early autism symptoms. *Journal of Child Psychology Psychiatry* 2011; 52(7): 741-52.

21. Vismara LA, Young GS, Rogers SJ. Telehealth for expanding the reach of early autism. *Autism Res Treat*. 2012; 2012:121878. doi:10.1155/2012/121878.
22. Humphrey, N. & Squires, G. Achievement for All national evaluation: final report. *Nottingham: DFE Publications* 2011.
23. Lendrum, A, Barlow,A. & Humphrey N. Developing positive school-home relationships through structured conversations with parents of learners with special educational needs and disabilities (SEND). *Journal of Research in Special Educational Needs. Early View* 2013; 1-9
24.Barrett B, Byford S, Sharac J, Hudry K, Leadbitter K, Temple K, Aldred C, Slonims V, Green J, the PACT Consortium. Service and wider societal costs of very young children with autism in the UK. *Journal of Autism and Developmental Disorders* 2012; 42(5): 797-804.
25. Gotham K, Pickles A, Lord C. Standardizing ADOS Scores for a Measure of Severity in Autism Spectrum Disorders. J Autism Dev Disord. 2009;39:693-705.
26. Pickles A, Le Couteur A, Leadbitter K, Salomone E, Cole-Fletcher R, Tobin H, Gammer I, Lowry J, Vamvakas G, Byford S, Aldred C, Slonims V, McConachie H, Howlin P, Parr JR, Charman T, Green J. Parent-mediated social communication therapy for young children with autism (PACT): long-term follow-up of a randomised controlled trial. *The Lancet*, 2016; 388, 2501-2509.
27. Estes A, Munson J, Rogers SJ, Greenson J, Winter J, Dawson G. Long-term outcomes of early intervention in 6-year-old children with autism spectrum disorder. *J Am Acad Child Adolesc Psychiatry*. 2015;54:580-7.
28. Carr T, Colombi C, MacDonald M, & Lord, C. Measuring respond to intervention with the Autism Diagnostic Observation Schedule-Change (ADOS-C). *Poster presented at Society for Research in Child Development Biennial Conference, Montreal, Canada 2011.*
29. Colombi C, Carr T, MacDonald M, & Lord, C. Developing a measure of treatment outcomes: The Autism Diagnostic Observation Schedule-Change. *Poster Presented at Society for Research in Child Development Biennial Conference, Montreal, Canada* 2011.
30. Grzadzinski R, Carr T, Colombi C, McGuire, K, Dufek S, Pickles A, Lord C. Measuring Changes in Social Communication Behaviors: Preliminary Development of the Brief Observation of Social Communication Change (BOSCC). *Journal of Autism and Developmental Disorders*. 2016; 46(7): 2464–2479.
31. Kitzerow J., Zok, V., Freitag C., Wilker C., Teufel K., Wilker C. et al. Using the Brief Observation of Social Communication Change (BOSCC) to measure autism-specific effects of an early intervention program. *Poster presentation. Enhancing the Scientific Study of Early Autism. EU COST conference.* September 2014. Toulouse, France.
32. Jones CR, Happé F, Pickles A, Marsden AJ, Tregay J, Baird G, Simonoff E, Charman T. 'Everyday memory' impairments in autism spectrum disorders. *Journal of Autism & Developmental Disorders*. 2011;41(4):455-64.
33. Mullen EM. Mullen scales of early learning. *Minnesota: American Guidance* 1995.
34. Elliot C D, & Smith P. British Abilities Scale-3 (BAS-3). *NFER-Nelson, Windsor, Berks, England* 2011.
35. Rutter M, Bailey A, Lord C. Social communication questionnaire. Los Angeles: Western Psychological Services, 2003.
36. Pickles A, Le Couteur A, Leadbitter K, Salomone E, Cole-Fletcher R, Tobin H, Gammer I, Lowry J, Vamvakas G, Byford S, Aldred C, Slonims V, McConachie H, Howlin P, Parr JR, Charman T, Green J. Parent-mediated social communication therapy for young children with autism (PACT): long-term follow-up of a randomised controlled trial. *The Lancet*, 2016; 388, 2501-2509.
37. Sparrow S S CDV, & Balla D A. Vineland adaptive behavior scales. *2nd ed. Oxford: NCS Pearson, Inc*; 2005.
38. Fenson, L., Marchman, V., Thal, D., Dale, P., Reznick, S., & Bates, E. (2007). MacArthur Communicative Development Inventories: User's guide and technical manual (2nd ed.). Baltimore, MD: Brookes.
39. Brownell R, ed. Receptive and expressive one-word picture vocabulary tests, 4th edn (ROWPVT-4, EOWPVT-4). San Antonio: Pearson Education, 2010.
40. Zimmerman, I. L., Steiner, V, G., & Pond, E. (2011). Preschool Language Scales- Fifth Edition (PLS-5). San Antonio, TX: Pearson.
41. Tennant R, Hiller L, Fishwick R, Platt S, Joseph S, Weich S, Parkinson J, Secker J, Stewart-Brown S. The Warwick-Edinburgh Mental Well-being Scale (WEMWBS): development and UK validation. *Health and Quality of Life Outcomes* 2007; 5:63.

42. Hatcher, R.L., & Gillaspy, J.A. (2006). Development and validation of a revised short version of the Working Alliance Inventory. Psychotherapy Research, 16, 12–25.
43. Parr, J.R., De Jonge, M.V., Wallace, S., Pickles, A., Rutter, M.L., Le Couteur, A.S., ... & Bailey, A.J. (2015). New interview and observation measures of the Broader Autism Phenotype: Description of strategy and reliability findings for the interview measures. Autism Research. Advanced online publication. doi: 10.1002/aur.1466.
44. Goodman R. The strengths and difficulties questionnaire: a research note. J Child Psychol Psychiatry 1997; 38: 581–86.
45. Kendall S. & Bloomfield L. (2005) Developing and validating a tool to measure parenting self-efficacy. J Adv Nurs, 51: 174–81.
46. Einfeld, S. L., & Tonge, B. J. (2002). Manual for the Developmental Behaviour Checklist: Primary Carer Version (DBC-P) & Teacher Version (DBC-T) (2nd. ed.). Clayton, Melbourne: Monash University Centre for Developmental Psychiatry and Psychology.
47. Stevens K. Valuation of the child health utility 9D index. Pharmacoeconomics. 2012;30:729–47.
48. Honey E, McConachie H, Turner M, Rodgers J. Validation of the repetitive behaviour questionnaire for use with children with autism spectrum disorder. Res Autism Sepctr Disord 2012; 6: 355–64.
49. StataCorp. College, Texas Stata statistical software: Release 13 [computer program]. *College Station: Stata Corp* 2013.
50. Kraemer HC. Discovering, comparing, and combining moderators of treatment on outcome after randomized clinical trials: a parametric approach. *Statistics in Medicine* 2013; 32(11):1964-73.
51. Emsley RA, Dunn G, White IR. Mediation and moderation of treatment effects in randomised controlled trials of complex interventions. *Statistical Methods in Medical Research* 2010; 19(3): 237-70.

Table S11 **PACT-G therapy fidelity rating scale**

**A. General Therapeutic Procedures**	
1. Review of Home/School Practice	
0	The therapist did not review the home/school programme or the parent/LSA's progress with the communication goals set.
1	The therapist asked the parent/LSA to recall communication goals set in the last session and written in the home/school programme, and reviewed the parent/LSA's progress with the goals in the home/school practice sessions.
2. Use of Video Excerpts to Illustrate Parent/LSA Achievement of their Goals	
0	The therapist did not show appropriate positive excerpts to illustrate achievement of parent/LSA communication goals, or selected excerpts and made comments on the parent/LSA-child communication that were vague or not related to the communication targets set.
1	The therapist showed appropriate video excerpts highlighting parent/LSA achievement of the communication targets set and elicited positive parent/LSA comments on change in parent/LSA communication style and child response.
3. Use of Video Excerpts as the Basis of Feedback Discussions	
0	The therapist did not adequately use positive video clips to promote discussion of the parent/LSA-child interaction, the focus of the stage and the strategies, AND/OR focused too much on negative clips rather than positive ones.
1	The therapist used appropriate positive video clips to facilitate parent/LSA reflection on the parent/LSA-child interaction, leading to the discussion of stage specific strategies and the focus of the stage.
4. Eliciting Parent/LSA Feedback following the Video Playback	
0	Limited therapist use of probes to elicit parent/LSA feedback. Did not use enough cascading probes to encourage the parent/LSA to reflect on the interaction and her/his role in it, or to be sure the parent/LSA understood the communication strategies and concepts being discussed.
1	The therapist elicited sufficient feedback from the parent/LSA. Cascading probes were effectively used to encourage parent/LSA reflection on the interaction and to determine the parent/LSA's understanding of the communication strategies and concepts under discussion.
5. Response to Parent/LSAs Focus	
0	No attempt to recognise or respond to parent/LSA's own observations and descriptions.
1	The therapist recognised and responded appropriately to parent/LSA's own descriptions of the interaction when reviewing the video; the therapist used the parent/LSA's own vocabulary and language to describe observations and strategies.
6. Structuring the Session	
0	Little or no structure to the therapy time and/or there were significant peripheral or unproductive digressions that were not handled well by the therapist.
1	The therapist structured session well, so there was a clear beginning, middle and closing of the session. Peripheral and unproductive digressions were either very uncommon, or handled well by the therapist.
7. Pacing	
0	The therapist's pacing and timing was either too rushed or not appropriately adapted to the parent/LSAs pace. The therapist may have moved on too quickly to another goal, giving the parent/LSA insufficient time to reflect and describe the changes in observed interaction or parent/LSA-child responses.
1	The therapist's pacing and timing was appropriately adapted to the parent/LSA's pace, giving sufficient time for the parent/LSA to reflect, participate and contribute observations to the discussion about the videoed interaction.
**B. Interpersonal Effectiveness**	
8. *Sensitivity Skills*	
0	The therapist did not sufficiently reflect or rephrase what the parent/LSA explicitly said, or showed problems responding to implicit or subtle parent/LSA communication. The therapist missed opportunities to respond to the parent/LSA's comments or responses during feedback.

1	The therapist generally seemed to grasp the parent/LSA's meaning as reflected by both what the parent/LSA explicitly said and what the parent/LSA communicated more subtly, and re-capped appropriately reflecting the parent/LSA's comments or descriptions.
9. Validation and Positive Feedback	
0	The therapist missed opportunities to reinforce or praise parent/LSA achievements and insights throughout the session.
1	The therapist recognised and appropriately reinforced or praised parent/LSA achievements and insights throughout the session.
**C. Specific Communication Therapy Techniques (score appropriate stage only)**	
10.1 Focus on shared attention (use for PACT-G stage 1)	The emphasis of this stage is on the development of shared attention between the parent/LSA and child.
0	The therapist did not adequately focus on the concept of shared attention and the stage 1 strategies that promote it, and/or the discussion of stage 1 strategies was not adequately linked to the development of shared attention.
1	There was adequate focus on the concept of shared attention. Appropriate stage 1 strategies were discussed and were adequately linked to the resultant changes in child responses and the development of shared attention.
10.2 Focus on parent/LSA synchronous communication style (use for PACT-G stage 2)	The focus of stage 2 is the development of parent/LSA's synchronous communication. The aim is to decrease parent/LSA's verbal demands on the child and replace these with comments aimed at facilitating and sustaining the child's communication responses. The parent/LSA's non-verbal responses and language become synchronous with the actions and intentions of the child.
0	There was not adequate focus on the concept of synchrony and the impact of the parent/LSA's communication style on the child's responses, AND/OR Stage 2 strategies were discussed but not adequately linked to the resultant responses of the child and the concept of synchrony.
1	There was adequate focus on the impact of the parent/LSA's communication style on their child's responses. Types of communication which elicited increased responses in the child were identified and discussed. Stage 2 strategies were appropriately introduced and adequately linked to the concept of parent/LSA synchrony.
10.3 Focus on Language Input (use for PACT-G stage 3)	Stage 3 focuses on ensuring that the language input the parent/LSA is giving the child maximises the child's opportunity to understand what they are hearing and to develop their language comprehension.
0	Lack of adequate focus on the identification of language mapping/ modelling opportunities and/or discussion of the appropriate level of language complexity for the child, AND/OR Stage 3 strategies introduced but not adequately linked to the development of the child's comprehension.
1	Therapist focuses session on the identification of opportunities for language mapping/modelling and/or discussion on the appropriate level of language complexity to use with the child. Stage 3 strategies are appropriately introduced and linked to the child's developing understanding of language.
10.4 Focus on predictable routines (use for PACT-G stage 4)	Stage 4 focuses on the parent/LSA using consistent, repetitive, familiar language routines and social routines commensurate with the child's level of language understanding. This is a phase of consolidation of all the preceding stages covering the use of repetition in play, interaction, language (e.g. repetitive rhymes, play themes, interactive routines). It also acts as a bridge to later stages, setting up predictable language routines in which the child may use their expressive language as it develops.
0	Lack of adequate feedback on the parent/LSA's use of consistent language, routines and play. Lack of adequate linking of the use of repetition to facilitate child verbal understanding and to set up predictable interactions in which the child may begin to use their expressive language in later stages.
1	Therapist focuses the session on identifying and encouraging the parent/LSA's appropriate use of consistent language, familiar routine and play. The therapist identifies opportunities for consolidation and elicits parent/LSA understanding of the way repetition facilitates child comprehension and processing, and may provide opportunities for child expression in later stages.
10.5 Focus on Communication initiation techniques (use for PACT-G stage 5)	Stage 5 focuses on the parent/LSA eliciting child intentional communication acts. The parent/LSA purposely uses pause and openings for the child to fill with a non-verbal or verbal response and includes the use of subtle teasing. This stage extends to the therapist facilitating the parent/LSA in

	eliciting child initiation for a range of communication functions, seeking/ directing adult attention, requesting, negating, acknowledging.
0	No or very limited discussion of techniques or games parent/LSA can use to create opportunities for child communication initiation, AND/OR No or limited discussion of the range of communication functions the child uses/could develop.
1	Therapist focused the session on identifying opportunities for child communication initiation and on discussion of techniques and games the parent/LSA can use to facilitate this. The therapist adequately discussed the range and function of the child's communication responses and initiations.
10.6 Focus on language extension, elaboration and reciprocal conversation (for PACT-G stage 6) Stage 6 focuses on the therapist assisting the parent/LSA to elaborate on and expand the child's expressive language repertoire i.e. finding opportunities to expand their utterances with semantically contingent information. The aim of this stage is to develop and extend child semantics and syntax. The therapist should also facilitate the parent/LSA in reciprocating in verbal interactions to develop mini-conversations that constitute at least 4 element conversational turns.	
0	The therapist does not adequately focus on identifying opportunities for parent/LSAs to extend and elaborate on the child's language and does not adequately discuss techniques for doing this. The therapist's attempts to identify opportunities for verbal reciprocal interchanges between parent/LSA and child were incomplete or inadequate.
1	Therapist focused the session on identifying opportunities and discussing techniques to extend and elaborate the child's language. An adequate variety of child language extension techniques and opportunities for verbal reciprocal conversations were identified with the parent/LSA.
11. Setting of mutually agreed goals for the Home/School Programme (PACT-G all stages)	
0	Goals were assigned with limited collaboration or were vague or incomplete, AND/OR goals set did not reflect the discussions in the session.
1	There was adequate setting of mutually agreed goals that included specific communication targets arising from the parent/LSA's observations, and insights achieved during the feedback session.
12. Written Home Programme (PACT-G all stages)	
0	No written goals in the home/school programme, or goals were unclear and/or not written in words the parent/LSA uses or understands AND/OR sections summarising the achievements in the session and giving practical examples of goals were not adequately completed.
1	Goals set in the session were written clearly in the home/school programme, in words the parent/LSA uses/understands. The home/school programme adequately summarised the achievements in the session and gave ideas of how the parent/LSA might practice the goals at home/school.
13. Deviation from Manual Were there any other deviations from the standard approach measured by this scale? If so do you think they were justifiable? (Do not include anything already scored)	
0	There were deviations and they were not justifiable (explain below)
1	There were no deviations OR any deviations were justifiable (explain below)
Details of Deviations:	
**D. Overall Ratings and Comments**	
14. Appropriate Use of Materials	
0	The therapist's selection of materials did not facilitate interaction.
1	The therapist's selection of materials did facilitate interaction.
15. Room Environment Did the room setting meet the requirements set out in the Standard Operating Procedures?	
0	No
1	Yes
16. Quality Time Was there adequate opportunity for parent/LSA- therapist discussion?	

0	No
1	Yes

PACT-G Fidelity Rating: adapted for use in PACT-G by Dr Catherine Aldred, Prof Jonathan Green from the Cognitive Therapy Scale (Jeffrey Young & Aaron Beck, modified by Leech, Harrington and Dubicka, 2002).
